# Rhizobacterial species richness improves sorghum growth and soil nutrient synergism in a nutrient-poor greenhouse soil

**DOI:** 10.1038/s41598-020-72516-3

**Published:** 2020-09-22

**Authors:** Mohammad Radhi Sahib, Zahida H. Pervaiz, Mark A. Williams, Muhammad Saleem, Seth DeBolt

**Affiliations:** 1 Department of Horticulture, University of Kentucky, Lexington, KY 40546-0312 USA; 2 Department of Horticulture, Al-Qasim Green University, Babylon, Iraq; 3 Department of Biological Sciences, Alabama State University, Montgomery, Al 36101 USA; 4 Department of Biological Sciences, Auburn University, Auburn, Al 36101 USA

**Keywords:** Biotechnology, Ecology, Microbiology, Plant sciences, Biogeochemistry, Ecology, Environmental sciences

## Abstract

Although microbes influence plant growth, little is known about the impact of microbial diversity on plant fitness trade-offs, intraspecific-interactions, and soil nutrient dynamics in the context of biodiversity-ecosystem functioning (BEF) research. The BEF theory states that higher species richness can enhance ecosystem functioning. Thus, we hypothesize that rhizobacterial species richness will alter sorghum (*Sorghum bicolor* L.) growth, soil nutrient dynamics and interactions (antagonism or synergism) in a nutrient-poor greenhouse soil. Using six rhizobacterial species in a BEF experiment, we tested the impact of a species richness gradient (0, 1, 3, 5 or 6 species per community) on plant growth, nutrient assimilation, and soil nutrient dynamics via seed-inoculation. Our experiment included, one un-inoculated control, six rhizobacterial monoculture *(Pseudomonas poae, Pseudomonas sp., Bacillus pumilus., Pantoea agglomerance., Microbacterium sp.,* and *Serratia marcescens*)*,* and their nine mixture treatments in triplicate (48). Rhizobacterial species richness enhanced per pot above- or below-ground dry mass. However, the per plant growth and plant nutrient assimilation declined, most likely, due to microbial-driven competitive interactions among sorghum plants. But nevertheless, some rhizobacterial monoculture and mixture treatments improved per plant (shoot and root) growth and nutrient assimilation as well. Soil nutrient contents were mostly lower at higher plant-associated rhizobacterial diversity; among these, the soil Zn contents decreased significantly across the rhizobacterial diversity gradient. Rhizobacterial diversity promoted synergistic interactions among soil nutrients and improved root–soil interactions. Overall, our results suggest that a higher rhizobacterial diversity may enhance soil–plant interactions and total productivity under resource limited conditions.

## Introduction

In present scenarios, agricultural intensification and anthropogenic activities are asserting a greater pressure on soil ecosystems while altering soil macro- and microorganisms^1–3^. Resultantly, a number of issues are emerging such as soil pollution, nutrient mining, erosion, aridity, and loss of soil microbial biodiversity ^1,4,5^. To address these issues, there is an emerging interest in applying ecological concepts to utilize microbial resources to improve soil fertility and plant growth ^5^. Considering the ecological and evolutionary significance of microbial biodiversity in the soil ecosystem ^6,7^, it is plausible that management and utilization of microbial resources could increase agricultural productivity in the marginal (i.e., agriculturally degraded or poor) soils. The most notable context is likely through the application of biodiversity and ecosystem functioning (BEF) theory in structuring the microbial communities for optimal plant growth. The BEF theory suggests that soil and plant systems inhabiting diverse microbial species may harvest more benefits from their microbial partners due to higher diversity and quantities of their beneficial properties ^5,8,9^. Thus, testing multiple combinations of the microbial species from monocultures to highly diverse species using a rigorous BEF experimental approach may help us screen and identify the high performing microbial consortia to develop probiotics for improving plant growth and yield in the marginal soils.

Meanwhile, the plant beneficial microbes are already in use for developing bio-fertilizers, plant growth stimulators, and bio-pesticides to reduce the use of agrochemicals. These microbes may enhance soil health, fertility, and plant growth by direct and indirect means, such as through, improving nutrition (nitrogen fixation, nutrient mobilization, recycling), regulating phytohormone (gibberellic acid, indole acetic acid, ethylene, and cytokinins, etc.) levels, suppressing soil-borne pathogens (via siderophores, cyanides, and antibiotics production), and ecological supportive roles ^10–12^. Owing to these properties, microbes may improve soil biochemical and ecological conditions while inducing tolerance in the plants against environmental stresses ^5,13,14^. Since the advent of next generation sequencing, the microbiome era has evolved into a resurgence of interest in understanding the role of microbial species diversity and composition in determining the soil health and plant productivity in broader ecological contexts ^15–18^.

Plants belonging to Panicoideae clade demonstrate excellent agro-ecological traits and are grown for fuel, fiber, and food production^19^. Among these, *Sorghum bicolor* L. (sorghum) is ranked as the world fifth major cereal crop after corn, wheat, rice and barley both in production and area covered ^20^. It serves as a staple food for many people all over the world, in addition, to playing a significant role in the economic and ecological stability in many countries^21^. Ecologically, it is a hardy drought tolerant C4 grass capable to thrive under resource limited conditions. By virtue of its ecological adaptation, sorghum plants exhibit symbiotic relationships with soil microbiota that determine their growth, survival, and/or tolerance to stress conditions ^22–24^. Some studies have, nevertheless, investigated sorghum-microbe interactions, mostly at individual microbial species/strain level, to test their significance in the sorghum growth under stress conditions ^25,26^. Sorghum is considered as a good crop for economic utilization in dryland cropping systems and marginal soils ^27–29^. Hence, it is timely to investigate sorghum-microbe interactions at community level to determine their role in the sorghum growth under resource limited soil conditions.

Though promising results are obtained from the soil, root and leaf inoculation studies ^11,30,31^, these approaches, however, are less feasible to adopt in the real-world agriculture due to the required amount of labor and technical skills at the farm level ^11^. In contrast, seed inoculation offers a potential advantage to harvest the benefits of applied probiotics due to technical ease ^32^. Given that seed germination is a major issue in obtaining the required plant density to get optimum yields from marginal soils^33^, seeding rates and resulting plant densities are widely debated with respect to their advantages and disadvantages. For instance, some studies have shown that high seeding rates and plant density may limit per capita production under benign conditions^34–38^. But nevertheless, there is a consensus that relatively higher plant density may provide greater agroecosystem services such as fodder or biomass production^36^, insurance against environmental stresses^37^, weed suppression ^39^, ground cover and erosion control ^40^. Therefore, we assume that microbial-driven differences in seed germination and resulting variation in plant density may lead to negative relationships (i.e., tradeoffs) between plant density (i.e., defined as the total number of plants per pot in our study) and per plant (PP) growth parameters (biomass, nutrient assimilation).

Given that nutrients availability is a microbial-driven process, we know little about the role of plant–microbe interactions in determining synergistic or antagonistic interactions among soil nutrients, a key parameter in soil health, fertility and plant growth ^41–44^. While most recent studies have not yet investigated the impact of synthetic communities on below-ground root properties, dynamics of soil macro-and micro-nutrients ^17,45^. The antagonistic interactions among soil nutrients are common under nutrient sufficient and deficient conditions, probably because of their competitive interactions and imbalanced composition during their uptake in the ionic forms^46^. The nutrient antagonism may potentially limit and restrict the uptake of soil nutrients by plants^47^. While antagonistic interactions among nutrients also depend, among others, on soil conditions, nutrient types, and crop cultivars^48–50^.The relatively high prevalence of these interactions under nutrient poor soil conditions may amplify the intensity of nutrient deficiency and limit plant growth ^46,48,49^. Though microbial effects on nutrient mobilization, fixation, and other soil properties are studied, a little is known about the effect of microbial biodiversity on nutrient-nutrient interactions in the soil environment.

Using BEF experimental approach, we tested the impact of microbial biodiversity on below/above ground *Sorghum bicolor* L. traits and nutrient interactions in a nutrient-poor greenhouse soil. We hypothesized that increasing the rhizobacterial species diversity of seed-inoculated rhizobacteria (i) may influence plant density due to its effect on seed germination followed by plant growth characteristics, tissue nutrient composition and trade‐offs among them while (ii) it may also affect the contents of soil macro-and micro-nutrients and interactions among them via plant density effect. Particularly, we investigated the effect of plant-bacteria partnership at higher diversity level on plant density, root as well as shoot system, plant and soil nutrient contents and their interactions. Subsequently, observing plant performance (shoot and root biomass) per pot, we anticipate the occurrence of trade-offs among plant traits and their relationships with nutrient contents of soil and plant tissues.

## Materials and methods

### Crop seeds

We used *Sorghum bicolor* L. cv Della in this study. The sorghum seeds were purchased from Townsends Sorghum Mill (Kentucky, USA). Prior to inoculation, seeds were washed and surface sterilized following the standard method. Briefly, prior to inoculation, the seeds were washed in the sterile deionized water (diH_2_O). Then, these seeds were surface-sterilized with a 20% household bleach solution for 10 min followed by three washes in the diH_2_O^51^.

### Rhizobacterial species

We used six rhizobacterial species in this study and these were derived from our lab collection. These species were isolated from the roots of switchgrass that was grown in two reclaimed sites in the Western Kentucky, USA (see details, ^52^). These six species included *Pseudomonas poae* A2S9 (XY10), *Pseudomonas sp.S16-2* (PSWZ), *Bacillus pumilus RC83* (UN4), *Pantoea agglomerance* GR13 (XY13), *Microbacterium sp., LKL04* (S23), and *Serratia marcescens PSB23* (R11). Here after, we refer these species as XY10, PSWZ, UN4, XY13, S23, and R11, respectively. All rhizobacterial species are sequenced and their genetic information is available elsewhere ^52^. We maintained rhizobacterial species on nutrient-rich organic medium (yeast extract/peptone/dextrose-YPD), thus implying that these bacteria are not obligate endophytes.

### Bacterial inoculation and development of a bacterial biodiversity gradient

All bacterial species were grown in the YPD broth medium at 29 °C and 200 rpm in a rotary shaker until the mid-log phase (optical density-OD_600nm_ = 0.2). All rhizobacterial inoculation treatments followed substitutive experimental design to develop a rhizobacterial biodiversity gradient^5^. Briefly, the starting density of inoculum for both monoculture and mixture treatments was kept same (OD_600nm_ = 0.2). For monoculture inoculation treatments, we inoculated sorghum seeds with 30 ml bacterial cultures (OD_600nm_ = 0.2) in the YPD broth medium using 50-ml Falcon plastic tubes. For 3-species mixture treatments, we took 10 ml of each bacterial culture (OD_600nm_ = 0.2), and then mixed them together to make a total volume of 30 ml solution in the 50-ml Falcon plastic tubes. For 5-species mixture treatments, we took 6 ml of each bacterial culture (OD_600nm_ = 0.2), and then mixed them together to make a total volume of 30 ml solution in the 50-ml Falcon plastic tubes. For 6-species mixture treatments, we took 5 ml of each bacterial culture (OD_600nm_ = 0.2), and then mixed them together to make a total volume of 30 ml solution in the 50-ml Falcon plastic tubes. By using this approach, we developed the initial rhizobacterial species richness or diversity gradient. The control seeds were treated with same volume of sterile YPD broth medium before sowing. Despite some variations, it took about 4 h to reach inoculated bacterial cultures to reach at the required density (OD_600nm_ = 0.6). Then, we took seeds out for sowing. We assumed that bacterial cultures might have colonized seeds effectively at this density (OD_600nm_ = 0.6). Other than keeping the same initial total density of bacterial monoculture and mixture treatments, we are not aware of, or, we did not take into account the initial possible differences in the individual bacterial biomass in the mixture treatments. Both inoculated and control sorghum seed were sown into plastic pots (10–12 ~ seeds per pot) containing greenhouse soil (Pro-Mix, Premier Horticulture Inc., PA, Quakertown, USA) while leaving ~ 5 cm at the top for proper aeration and drainage. The soil was steamed (to kill any existing pests or pathogens) for one hour before filling into pots. The experimental pot dimensions were as follow: height = 9 1/8"(~ 23 cm), diameter = 10 1/8"(26 cm), volume = 2.3 gallons (trade size = 2.5 gallon or 9.5 L). We conducted experiments in the greenhouse of University of Kentucky that was set for the standard sorghum growth conditions (16:8 light dark regime, 28 °C) ^52^. We watered plants from bottom using undertrays, as needed. The plants were twice fertilized with 500 ml fertilizer solution (20 N: 10 P: 20 K with a concentration of 200 ppm) from bottom using undertrays, immediately after, and after two weeks of germination. Following completely randomized design, the experimental treatments included; un-inoculated (control) and six different monoculture inoculation treatments. Moreover, we have four 3-species (N4/S23/PSWZ, UN4/XY13/S23, XY10/XY13/R11, and PSWZ/R11/XY10) mixture inoculation treatments. In addition, we have four 5-species (S23/R11/XY10/UN4/PSWZ, S23/R11/XY10/XY13/PSWZ, UN4/R11S23/XY13/PSWZ, and UN4/R11/XY10/XY13/PSWZ) and one 6-species mixture treatments that included all bacterial species together. All control and inoculation treatments were in triplicates. The position of pots in the greenhouse was changed often to minimize the position effects ^30^.

### Data measurement and analysis

After 5 weeks, we harvested plants and took soil, root and shoot samples for further analysis. We manually counted total number of plants and root branches from each pot. Apart from measuring plant shoot and root biomass per pot, we also calculated shoot and root biomass per plant by dividing the shoot biomass per pot by plant density. After taking out roots from pots, we removed soil from roots by shaking and brushing. We immediately submitted soil samples to the soil lab in the Division of Regulatory Services at the University of Kentucky for the water-extractable/soluble soil nutrient analysis. The details of these methods can be accessed online (Methods: https://www.rs.uky.edu/soil/tests/methods.php). The below and above-ground portions of plants were oven-dried at 60 °C for 48 h before biomass measurements. Both root and shoot samples were ground to determine their N, P, and K contents, following standard methods^53–56^. The details about these methods are provided (see supplementary information). Unless otherwise stated, the concentrations of all soil nutrients were expressed as mg per liter of soil extract, except for soil N-nitrate contents (those were in ppm). While the N, P, and K concentrations of plant shoot tissues were expressed as percentage of total mass. Moreover, it is important to mention that we did not intend to compare these correlations in the rhizobacterial versus control treatment because later has just three replicates and any observation from these might be due to the statistical artifacts.

### Data analysis

**Figure 1:**
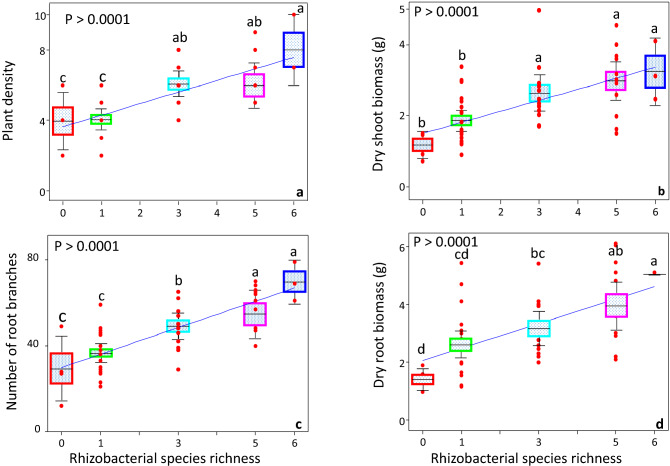
Impact of rhizobacterial species richness on the plant density (**a**), dry shoot biomass (**b**), number of root branches (**c**), and dry root biomass (**d**). Error bars represent means ± 1SE while lack of shared letters above the bars represents the significant differences among richness levels.

**Figure 2:**
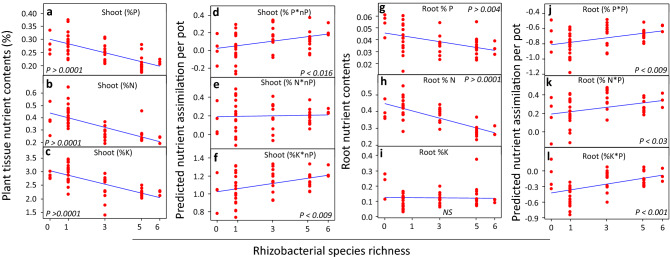
Impact of rhizobacterial species richness on shoot nutrient contents (**a**–**c**). Relationship between rhizobacterial species richness and predicted nutrient contents (actual nutrient contents multiplied by plant density) (**d**–**f**). Impact of rhizobacterial species richness on root nutrient contents (**g**–**i**). Relationship between rhizobacterial species richness and predicted root nutrient contents (actual nutrient contents multiplied by plant density) (**j**–**l**).

**Figure 3:**
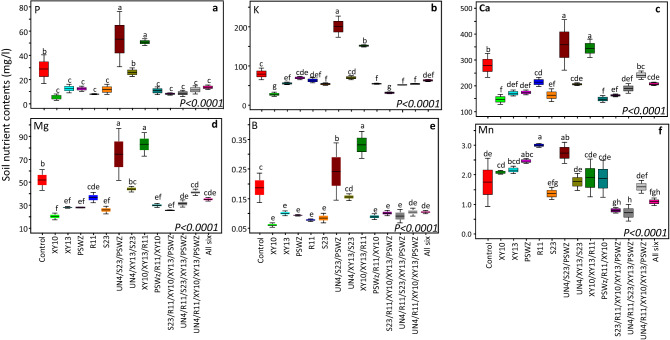
Impact of individual rhizobacterial species and their various combinations on soil nutrient contents. The error bars indicate means ± 1SE while the lack of shared letters above the bars represents the significant differences among control, monoculture, and mixture treatments. We don’t have data of one monoculture (UN4) and one 5-species mixture (S23/R11/XY10/UN4/PSWZ), and these are not plotted.

**Figure 4:**
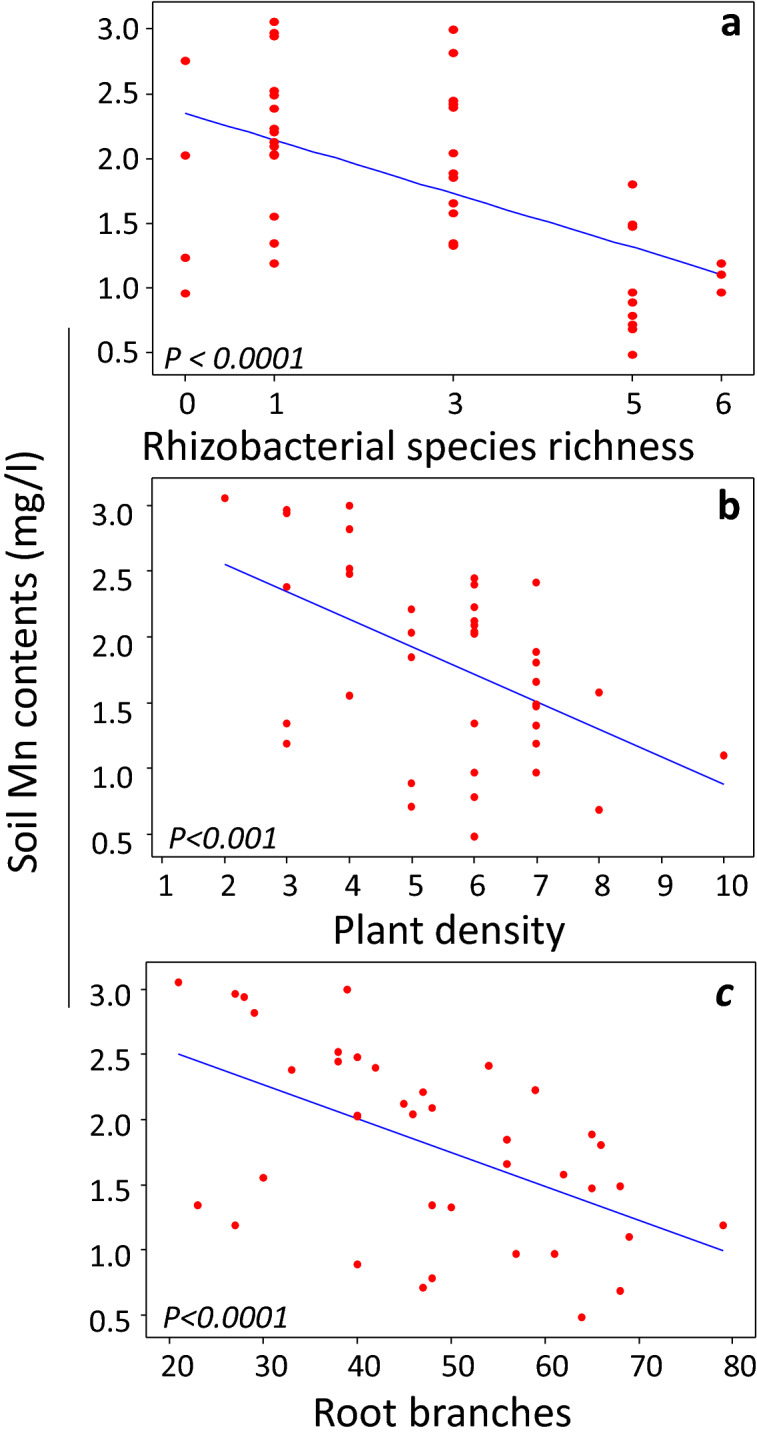
Impact of rhizobacterial species diversity on soil Mn contents (**a**). The relationships of plant density (**b**) and root branches (**c**) with Mn contents in the rhizobacterial treatments.

**Figure 5:**
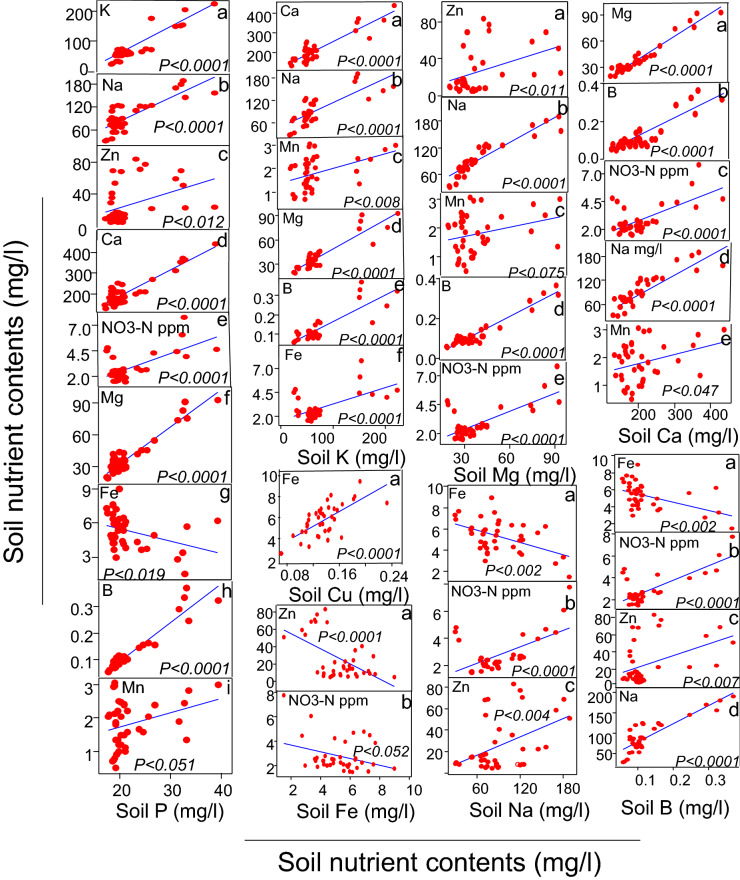
Relationships between contents of various soil macro-and micro-nutrients in the rhizobacterial treatments.

**Figure 6:**
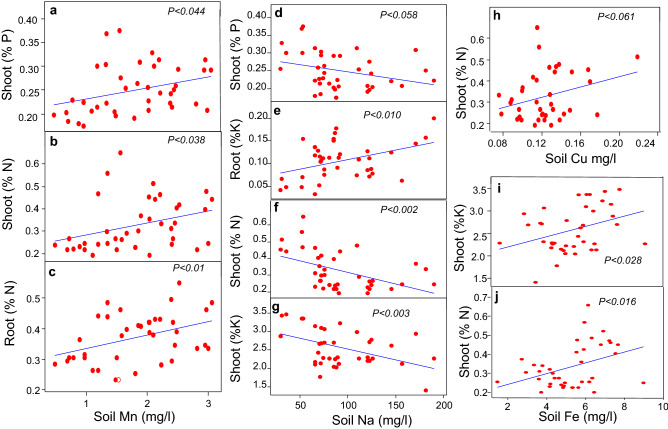
Significant relationships of soil nutrient contents with plant tissue nutrient contents in the rhizobacterial treatments.

We conducted different statistical tests to perform data analysis. The general linear-regression analysis was performed to determine the relationship of rhizobacterial species richness with plant growth parameters, such as plant density, dry shoot biomass, number of root branches, and dry root biomass. Each richness level represents the data points of all replicates of control, bacterial monoculture, and mixture combinations. Moreover, we also performed ANOVA followed by Fisher's post hoc test to determine significant differences among plant densities across different rhizobacterial richness levels (Fig. 1a–d). Using general linear-regression analysis, we also determined the relationship of rhizobacterial species richness with shoot (Fig. 2a–c) and root (Fig. 2 g–i) nutrient contents. Similarly, using general linear-regression analysis, the relationship of rhizobacterial species richness with predicted total shoot (Fig. 2d–f) and root (Fig. 2j–l) nutrient contents (actual nutrient contents multiplied by plant density) was also determined. The predicted nutrient assimilation per pot (Fig. 2d–l) does not imply any quantitative prediction or claim rather it is a qualitative prediction of nutrient assimilation by all plants in the pots. Furthermore, we also determined the differences in soil nutrient contents in control, rhizobacterial monoculture, and mixture treatments by ANOVA followed by the Fisher's post hoc test (Fig. 3a–f). Using general linear-regression analysis, the relationship of rhizobacterial species richness, plant density, and root branches per pot (plant density) with soil Mn contents was determined (Fig. 4a–c). Similarly, the relationships between contents of various soil macro-and micro-nutrients in the rhizobacterial treatments were determined by general linear-regression analysis (Fig. 5). Moreover, the relationships of soil nutrient contents with plant tissue nutrient contents in the rhizobacterial treatments were also determined by general linear-regression (Fig. 6). The details about analysis of supplementary figures are described in their corresponding legends (see supplementary information).

## Results

### Impact of rhizobacterial diversity on above-ground plant performance

#### Plant density

The plant performance varied in response to the different rhizobacterial species (Fig. 1, Fig. S1). Generally, the rhizobacterial monoculture treatments had a non-significant impact on plant density. Interestingly, seeds inoculated with three, five and/or six rhizobacterial species mixtures, exhibited greater plant density due to better seed germination than control plants. Overall, rhizobacterial species richness increased the plant density (Fig. 1a, Fig. S1).

#### Shoot biomass per pot and per plant

Contrary to the plant density, some rhizobacterial monocultures (XY13, PSWZ) increased plant shoot biomass per pot relative to the control treatments (Fig. S2a). On average, rhizobacterial 3-species mixture inoculation also increased shoot biomass as compared to the control. Similarly, five- and six-species mixture inoculation also enhanced shoot biomass per pot than control plants. However, two five-species mixtures, such as S23/R11/XY10/UN4/PSWZ and S23/R11/XY10/XY13/PSWZ treatments, showed lower shoot biomass, as compared to, other 5- and a 6-species mixture treatments in terms of their positive effects on shoot biomass (Fig. S2a). While in general, an increase in the seed-associated rhizobacterial species diversity via inoculation demonstrated a significant positive effect on the shoot biomass (Fig. 1b). Contrarily, we observed a weak effect of rhizobacterial inoculation on per plant shoot biomass; only one rhizobacterial monoculture (PSWZ) and a 5-species mixture (UN4/R11/S23/XY13/PSWZ) increased shoot biomass per plant (Fig. S2d). Thus, measuring per plant biomass helped us determined the tradeoff between total pot productivity and per plant performance. We observed a substantial tradeoff between plant density and per plant shoot biomass (Fig. S3a, S4a). These data suggested that per pot productivity increased, however, mostly per plant shoot biomass did not increase significantly under rhizobacterial influence (Fig. S3a, S4a).

#### Nutrient assimilation by shoots

The N than both P and K uptake by plant shoots was relatively higher (Fig. 2). In general, the nutrient contents of plant shoots in the mixture- inoculation treatments were lower than those in the monoculture or control treatments (Fig. S5a,c,e). Nevertheless, the plants growing under the influence of one monoculture (S23) treatment showed relatively higher N and P contents (Fig. S5a,c,e). Interestingly, the plant density exhibited a significant negative relationship with the plant shoot nutrient contents (Fig. S6-7a,b,c). Moreover, we also found a negative relationship between total shoot biomass per pot and the soil nutrient contents (Fig. S6-7d–f). However, we did not find any relationship between shoot biomass per plant and soil nutrient contents (Fig. S6-7 g–i). Overall, the assimilation of nutrients decreased across the rhizobacterial seed-inoculation diversity gradient (Fig. 2a–c); however, multiplying the plant density with their tissue nutrient contents predicted a positive impact of rhizobacterial seed-inoculation diversity gradient on total plant nutrient (except N) assimilation per pot (Fig. 2d–f).

### Impact of rhizobacterial diversity on belowground plant performance

#### Root traits per pot and per plant

The rhizobacterial seed-inoculation significantly influenced the root system of sorghum plants, especially in terms of root branches and biomass per pot. In this regard, two monoculture treatments such as XY10, and XY13 significantly increased the number of root branches per pot than control treatment. Likewise, most 3- (except UN4/S23/PSWZ), 5-, and 6-species mixture treatments showed significantly more root branches per pot than control treatment (Fig. S2b). Contrary to per pot root branches, per plant root branches increased in some monoculture treatments (PSWZ, UN4, and R11). A non- significant increase in the root branches was observed in the 3-, 5-, and 6-species mixture treatments, however, this effect was statistically significant in two 5-species mixtures (*UN4/R11/S23/XY13/PSWZ* and *UN4/ R11/ XY10/XY13/PSWZ*) treatments (Fig. S2e). Furthermore, rhizobacterial treatments had a significant impact on root biomass per pot. In this regard, two monoculture (XY13 and PSWZ) and two 3-species mixture (UN4/S23/PSWZ and UN4/XY13/S23) treatments significantly increased root biomass per pot than control treatment (Fig. S2c). Mostly, 5- (except S23/R11/XY10/XY13/PSWZ) and 6-species mixture treatments increased root biomass per pot than control treatment (Fig. S2c). Interestingly, some monoculture such as PSWZ, R11 and UN4 treatments showed a significant effect on the root biomass per plants than control treatment (Fig. S2f.). Overall, the rhizobacterial seed-inoculation resulted into interesting tradeoffs between per pot and per plant root branches and root biomass. Particularly, plant density always negatively correlated with per plant root biomass and branches (Fig. S3-4 b,c). However, at the per pot level, rhizobacterial diversity was correlated with increased root biomass and branches in the greenhouse soil (Fig. 1c,d).

#### Nutrient assimilation by roots

The plants growing under the influence of one monoculture treatment, namely, PSWZ, showed higher root N content; however, N consistently decreased across the rhizobacterial seed-inoculation diversity gradient (Fig. S5b). Similarly, trends were observed in cases of P and K assimilation in the plant roots (Fig. S5d,f). Overall, N and P (except K) contents decreased across the rhizobacterial seed-inoculation diversity gradient (Fig. 2 g–i). However, if we multiply the plant density with root tissue nutrient content, then our results predicted a positive impact of the rhizobacterial seed-inoculation diversity gradient on the assimilation of nutrients by the plant roots (Fig. 2j–l). Furthermore, we also investigated the relationship between plant density and root nutrient composition. Interesting, the plant density exhibited a negative relationship between nutrients (P, N) and the plant root tissues (Fig. S6-7 j–l). While, only N contents negatively correlated with per pot root dry mass (Fig. S6-7 m–o). But, per plant dry root mass positively correlated with root N (Fig. S6-7p–r).

### Impact of rhizobacterial-seed inoculation diversity gradient on soil micro-and macro-nutrients

#### Impact of rhizobacterial monoculture and mixtures treatments on soil macro- and micronutrients

The soil P was significantly lower in the rhizosphere of plants that grew from seeds inoculated with monocultures, one 3-, (PSWZ/R11/XY10), all 5-, and 6-species mixtures (Fig. 3a). Similarly, K, calcium (Ca), magnesium (Mg), and Boron (B) contents were also lowest in the same treatments (Fig. 3). Among soil nutrients, the Mn contents showed interesting patterns under different treatments. Though soil Mn contents were relatively higher in the pots in the monoculture and most mixture treatments, however, they tended to decline in the 5-, and 6-species mixtures. The monoculture namely, S23, showed non-significantly lower soil Manganese (Mn) contents than control treatment (Fig. 3f). Interestingly, the soil Mn contents decreased linearly across the rhizobacterial seed-inoculation diversity gradient (*P* < *0.0001)* (Fig. 4a). Moreover, the soil Mn contents significantly declined with an increasing number of plants (*P* > *0.002*) and root branches (*P* > *0.001*) per pot (Fig. 4b,c, Fig. S9a,b). Although, we observed a poor impact of rhizobacterial inoculation on Copper (Cu) uptake, but still, the soil Cu contents were lower in the monocultures, one 3-, all 5-, and 6-species mixture treatments (Fig. S8a). The soil sodium (Na) contents were significantly lower in the monocultures, one 3-, (*PSWZ/R11/XY10*), most 5-, and 6-species mixture treatments (Fig. S8c). However surprisingly, most 3-species mixture (i.e., UN4/S23/PSWZ, UN4/XY13/S23, and XY10/XY13/R11) than control treatments showed relatively higher contents of soil P and other nutrients such as K, Ca, Mg, B, Na, etc. (Fig. 3, Fig. S8). Interestingly, opposite to the uptake of above-mentioned nutrients, the soil iron (Fe) content was significantly lower in the 3-species mixture (UN4/XY13/S23, XY10/XY13/R11, PSWZ/R11/XY10) than control treatments. In contrast, the soil Fe in a monoculture (XY10) and 6-species mixture were significantly higher than control and other treatments (Fig. S8). One monoculture (XY10), and two 3-speciesmixture (UN4/S23/PSWZ, XY10/XY13/R11) treatments showed higher than control soil N (N-NO_3_) contents. The soil Zn contents were lower in the monocultures, one 3-species (PSWZ/R11/XY10), all 5-, and 6-species mixture treatments (Fig. S8e). However surprisingly, in three cases, two monocultures (PSWZ, R11), and three 3-species mixtures showed significantly higher soil Zn contents than those in the control and other treatments (Fig. S8e).

#### Interactions among soil nutrients

We also investigated the relationships between soil nutrients. The soil P showed strong positive relationships with soil Mn, B, Mg, N (NO_3_ ppm), Ca, Na, and K. The opposite was true for it’s relationship with soil Fe (Fig. 5). Similarly, soil K contents showed positive relationships with soil Fe, B, Mg, Mn, Na, and Ca contents. The soil Cu contents showed strong positive relationship with the soil Fe contents. While soil Fe contents showed strong negative relationship with soil N (NO_3_ ppm) and Zn contents. Similarly, soil Mg contents showed positive relationships with soil N (NO_3_ ppm), B, Mn, Na, and Zn contents. The soil Na contents showed positive relationship with Zn and N (NO_3_ ppm) contents while opposite is true for its relationship with the soil Fe contents. Soil Ca contents demonstrated strong positive relationships with the soil Mn, Na, N (NO_3_ ppm), B, and Mg contents. While soil B contents showed strong positive relationships with soil Na, Zn, and N (NO_3_ ppm) contents while converse is true for its relationship with the soil Fe contents (Fig. 5, Fig. S10). Overall, most soil macro- and micro-nutrient contents showed positive relationships with each other. As described above, the soil Mn contents responded strongly to the rhizobacterial seed-inoculation gradient while Mn contents also showed strong positive relationships with both soil macro-and micronutrients (Figs. 4, 5, Fig. S10).

#### Linking soil nutrients to plant tissues nutrients

The soil Na, Cu, Fe, and Mn exhibited strong relationships with the N, P, and K contents of plant tissues. The soil Mn contents showed significant positive relationships with root and shoot N contents while demonstrated same correlation with shoot P contents (Fig. 6a–c). The soil Na contents exhibited significant negative relationships with the P, K, and N contents of the plant tissues. While the soil N contents showed positive relationship with the root K contents (Fig. 6d–g). However, the soil Cu contents showed significant positive relationship with N contents of the plant tissues. Similarly, the soil Fe contents exhibited significant positive relationship with the shoot K and N contents (Fig. 6h–j).

## Discussion

Several studies have investigated the microbial associations with plants in agricultural and ecological settings in a broad range of soil conditions^12,57,58^. In the current study, we tested the impact of rhizobacterial species richness (via seed-inoculation) on plant performance traits and soil nutrient dynamics using a BEF experimental approach. We mapped these results against the metrics of plant growth performance traits including plant density, root and shoot system biomass, density, nutrient assimilation, tradeoffs among these traits, soil nutrient contents and their interactions with each other.

Previously, some studies have reported a positive effect of individual rhizobacterial species on plant density due to their positive effects on seed germination of sorghum and other plants ^59,60^; however, we did not find a significant positive effect of six different individual rhizobacterial species on this plant trait. While increasing the species diversity of rhizobacterial seed-inoculated probiotics substantially increased plant density (Fig. 1a, Fig. S1) that may suggest the role of diverse microbes in breaking the dormancy and increasing the seed germination and plant density. Other than this ecophysiological observation, our results don’t provide any molecular basis of an enhanced plant density across the rhizobacterial seed-inoculation gradient; however, it merits future research to discern the underlying interactions and mechanisms since both plants and microbes select each other at this stage.

A relatively better plant density in the rhizobacterial treatments increased per pot shoot biomass, root biomass and branches. The above-and below-ground per pot productivity was seen in the monocultures and mixtures treatments, though later demonstrated more significant impact on the plant productivity (Fig. 1b–d, Fig. S2a–c). This, nevertheless, confirm the recent predictions that an increase in microbial species diversity may increase the beneficial properties essential for host plant growths ^17,61^. As per our monoculture data, only few monocultures increased plant growth greater than control treatments. Most of the cases, monoculture effects were poor, thus suggesting that probiotics containing individual microbial species may not confer advantages to host plants either due to their relatively low survival and/or due to lack of multiple plant beneficial traits. But with increasing the species diversity of rhizobacterial probiotics, the plant growth responded dramatically that, nevertheless, predicted the teamwork of rhizobacterial species in the mixtures ^5,17,62^ (Fig. S2a–c). In addition, a relatively high above-and below-ground plant productivity as a function of species-rich probiotics may also imply that microbial associations with plants at higher diversity levels may increase plant productivity. However, increased plant density and intraspecific-competition among plants (for water, nutrients and other resources) may lead to some ecological costs under marginal soil conditions. For instance, per plant performance tended to decrease in the rhizobacterial than control treatments (Fig. S2d–f, S3-4a–c) while plant density correlated negatively with plant fitness traits such as shoot, root biomass and branches. The plant density is a key determinant of agro- and natural-ecosystems productivity while it influences the quality and quantity of important agro-ecological traits ^63–65^. Nevertheless, the higher plant density, as we observed in the rhizobacterial treatments, is considered a good indicator of plants agronomic traits and productivity under marginal soil conditions ^66,67^.

A negative relationship between plant density and per plant productivity was also translated to the nutrient contents of root and shoot tissues. Interestingly, most of the cases, rhizobacterial seed-inoculation diversity gradient, plant density, shoot and root biomass negatively correlated with the N, P, and K contents of plant tissues (shoots, roots). As described above, it is very likely that an initial microbial-driven enhanced plant density and total plant productivity caused intraspecific competition among sorghum plants. Some studies suggested that low per plant biomass and nutrient contents under poor soil conditions may reflect an intense intraspecific competition ^68,69^. The below-ground intraspecific competition may limit per plant productivity ^69,70^ whereas some recent studies have predicted the role of soil organisms in determining these interactions ^71^. Moreover, intraspecific competitions are common in grasses because they are considered superior competitors for nutrients ^72,73^ under nutrient limited soil conditions. Although sorghum plant–microbe partnership is reported, our results anticipate that microbial biodiversity may promote intraspecific competition among sorghum plants, and thus our prediction was supported by plant growth parameters (Fig. S3-4–5-6, Fig. 2) as well as root and shoot nutrient contents (Fig. 2). Meanwhile, our results suggest that developing any strategy to harness plant–microbe interactions for the restoration and economic utilization of marginal lands may require a precise assessment of plant intraspecific interactions to optimize the agro-ecological benefits ^68^.

Given higher plant density and per pot productivity in the rhizobacterial treatments, our results showed a corresponding greater uptake of macro-and micronutrients from the soil than control treatments. Most of past and recent studies have indeed showed a relatively greater assimilation of N, P, and K by the tissues of plants inoculated with microbes ^17,45^, however, the impact of plant-associated microbial diversity on soil nutrient contents are less explored (except for N fixation and solubilization studies)^74,75^. As a general trend, the soil nutrients P, K, Ca, Mg, B, Mn, Cu, Fe, Na, N (NO_3_ ppm) and Zn were lower in the rhizobacterial than control treatments, thus implying the role of plant-associated microbes in exploiting soil nutrients. Apart from N-fixation and nutrient (e.g. P, Fe) solubilization studies, limited information is available regarding the role of plant–microbe interactions in altering the soil macro- and micro-nutrients ^18,76–78^. Interestingly, in some cases, the soil nutrient contents were relatively high in the rhizobacterial treatments (e.g., relatively species poor 3-species mixture treatments) (Fig. 3, Fig. S8). This result may suggest that better nutrient uptake, assimilation and corresponding productivity is ensured by maintaining higher microbial diversity, as we have observed in 5–6 species mixture treatments. Thus, supporting the importance of a greater microbial species richness in delivering greater ecosystem services following the BEF theory ^5,8,79^. Meanwhile, as we observed in some monoculture treatments, it is also important to mention and acknowledge the role of some single-species probiotics in enhancing the plants' ability to exploit soil nutrients that is in line with classical agricultural (i.e., beneficial rhizobacterial species) and ecological (i.e., keystone or singular species concept) research emphasizing the significance of individual species in the plant growth and other agroecosystem processes ^80–82^.

Interestingly, among all soil nutrients, Mn responded significantly to the rhizobacterial inoculation-diversity gradient. Here, an increase in the rhizobacterial species richness linearly decreased the Mn content while the same relationship was observed with plant density and root branches. ^83,84^. Our results suggest that plants with dense or clustered roots can effectively exploit Mn from soils under nutrient-limited conditions ^85^. Despite being a limiting factor in plant growth and development, most plant–microbe studies have focused on phytoextraction and phytostabilization of Mn in the contaminated sites ^86,87^. The microbial-driven an enhanced below and above-ground plant density may not only lead to higher total biomass but may also increase Mn uptake from the soil. Other than microbial-mediated plant effects on the soil Mn contents, we did not investigate microbial mechanisms of Mn transformation, solubility, and availability to plants. By doing so, we may have an increased ability to understanding the microbial role in altering the soil Mn contents. But, we anticipate that plant–microbe partnership might help in the restoration of Mn or heavy metal-contaminated soils for multiple agro-ecological purposes, such as phytoremediation.

Most rhizobacterial taxa that we tested in this study are known to fix and cycle nutrients by solubilizing metal complexes (e.g., Fe–P, Ca–P, and Mn–P)^88^. Considering an enhanced plant growth and nutrient uptake as a function of rhizobacterial diversity, we also studied interactions among various soil nutrients. In general, antagonistic interactions among soil nutrients are widely reported in the soil fertility literature^89,90^. Using linear-regression analysis, we found that most of the case, soil nutrients showed strong positive relationships with each other in the bacterial treatments that nevertheless predicts a microbial-driven synergism among soil nutrients. As described before, for instance, soil Mn contents not only responded to seed-inoculated rhizobacterial diversity, root branches, and plant density (Fig. 4) but also showed positive relationships with several soil micro-and macro-nutrients (Fig. 5). Moreover, Mn contents also showed positive relationships with root N, shoot N and P contents (Fig. 6), thus predicting a strong synergism between soil and plant nutrient contents. The synergism among soil nutrients determines soil fertility, plant nutrition ^41,46,91^ and interactions with other species ^92^. Our results are the first report to describe the role of rhizobacterial diversity in causing synergism among soil nutrients that is an important ecosystem service underling plant productivity ^43,93^. Meanwhile, we also found some negative correlations between soil nutrients, for instance, the soil Fe negatively correlated with soil P, Cu, nitrate–N, B, Zn, and Na contents, thus suggesting that soil Fe contents are highly sensitive to the contents of other nutrients in the soil^94^.

Overall in this study, we attempted to forecast the impact of seed-inoculated rhizobacterial species richness on soil and plant systems in broader ecological contexts. It is plausible that introduced microbes may have led to an inherent organization of the microbial community in the rhizosphere and root system while the coordinate behavior of rhizobacterial species imparted significant changes to the plant traits and soil nutrient dynamics. As described earlier in this manuscript about several mechanisms that may underlie these changes; however, we did not study the underlying microbial beneficial properties. Our study primarily focused on the plant growth traits, tradeoffs and soil nutrient contents while our results corresponds with below ground and above-ground properties and nutrient assimilation. Our data predict a required balance between microbial competitiveness and plants productivity. Due to plant and soil properties being primarily influenced by the probiotic mixes, we suggest that further field trials in marginal lands may reveal further insights into plant–microbe interactions at higher diversity levels.

## Supplementary information

Supplementary Information 1.

Supplementary Information 2.

## References

[1] Liu Z (2020). Effect of simulated acid rain on soil CO2, CH4 and N2O emissions and microbial communities in an agricultural soil. Geoderma.

[2] Li M (2020). Biochemical response, histopathological change and DNA damage in earthworm (*Eisenia fetida*) exposed to sulfentrazone herbicide. Ecol. Indic..

[3] Zhang Q, Saleem M, Wang C (2019). Effects of biochar on the earthworm (*Eisenia foetida*) in soil contaminated with and/or without pesticide mesotrione. Sci. Total Environ..

[4] Wu Y (2020). Ecological clusters based on responses of soil microbial phylotypes to precipitation explain ecosystem functions. Soil Biol. Biochem..

[5] Saleem M, Hu J, Jousset A (2019). More than the sum of its parts: microbiome biodiversity as a driver of plant growth and soil health. Annu. Rev. Ecol. Evol. Syst..

[6] Fierer N (2017). Embracing the unknown: disentangling the complexities of the soil microbiome. Nat. Rev. Microbiol..

[7] Saleem M, Saleem M (2015). Ecoevolutionary processes regulating microbiome community assembly in a changing global ecosystem. Microbiome Community Ecology: Fundamentals and Applications.

[8] Loreau M (2001). Biodiversity and ecosystem functioning: current knowledge and future challenges. Science.

[9] Prosser JI (2007). The role of ecological theory in microbial ecology. Nat. Rev. Micro..

[10] Lugtenberg B, Kamilova F (2009). Plant-growth-promoting rhizobacteria. Annu. Rev. Microbiol..

[11] Bashan Y, Bashan LE, Prabhu SR, Hernandez J-P (2013). Advances in plant growth-promoting bacterial inoculant technology: formulations and practical perspectives (1998–2013). Plant Soil.

[12] Sun T, Li M, Saleem M, Zhang X, Zhang Q (2020). The fungicide “fluopyram” promotes pepper growth by increasing the abundance of P-solubilizing and N-fixing bacteria. Ecotoxicol. Environ. Saf..

[13] Dimkpa C, Weinand T, Asch F (2009). Plant–rhizobacteria interactions alleviate abiotic stress conditions. Plant Cell Environ..

[14] Sun T (2020). Bacterial compatibility and immobilization with biochar improved tebuconazole degradation, soil microbiome composition and functioning. J. Hazard. Mater..

[15] van Elsas JD (2012). Microbial diversity determines the invasion of soil by a bacterial pathogen. Proc. Natl. Acad. Sci..

[16] Delgado-Baquerizo M (2016). Microbial diversity drives multifunctionality in terrestrial ecosystems. Nat. Commun..

[17] Hu J (2017). Probiotic Pseudomonas communities enhance plant growth and nutrient assimilation via diversity-mediated ecosystem functioning. Soil Biol. Biochem..

[18] Woo SL, Pepe O (2018). Microbial consortia: promising probiotics as plant biostimulants for sustainable agriculture. Front. Plant Sci..

[19] Paterson AH (2009). The Sorghum bicolor genome and the diversification of grasses. Nature.

[20] USDA. Sorghum Production by Country | World Agricultural Production 2019/2020. https://www.worldagriculturalproduction.com/crops/sorghum.aspxhttps://www.worldagriculturalproduction.com/crops/sorghum.aspx (2019).

[21] Zhao Z-Y, Che P, Glassman K, Albertsen M, Zhao Z-Y, Dahlberg J (2019). Nutritionally enhanced sorghum for the arid and semiarid tropical areas of Africa. Sorghum: Methods and Protocols.

[22] Schlemper TR (2017). Rhizobacterial community structure differences among sorghum cultivars in different growth stages and soils. FEMS Microbiol. Ecol.

[23] Xu L (2018). Drought delays development of the sorghum root microbiome and enriches for monoderm bacteria. Proc. Natl. Acad. Sci..

[24] Hara S (2019). Identification of nitrogen-fixing bradyrhizobium associated with roots of field-grown sorghum by metagenome and proteome analyses. Front. Microbiol..

[25] Idris HA, Labuschagne N, Korsten L (2007). Screening rhizobacteria for biological control of Fusarium root and crown rot of sorghum in Ethiopia. Biol. Control.

[26] Idris A, Labuschagne N, Korsten L (2009). Efficacy of rhizobacteria for growth promotion in sorghum under greenhouse conditions and selected modes of action studies. J. Agric. Sci..

[27] Kort J, Collins M, Ditsch D (1998). A review of soil erosion potential associated with biomass crops. Biomass Bioenergy.

[28] Truong SK, McCormick RF, Mullet JE (2017). Bioenergy sorghum crop model predicts VPD-limited transpiration traits enhance biomass yield in water-limited environments. Front. Plant Sci..

[29] Li C (2017). Soil carbon sequestration potential in semi-arid grasslands in the Conservation Reserve Program. Geoderma.

[30] Saleem M, Ji H, Amirullah A, Brian Traw M (2017). Pseudomonas syringae pv tomato DC3000 growth in multiple gene knockouts predicts interactions among hormonal, biotic and abiotic stress responses. Eur. J. Plant Pathol..

[31] Zhang Q, Saleem M, Wang C (2017). Probiotic strain Stenotrophomonas acidaminiphila BJ1 degrades and reduces chlorothalonil toxicity to soil enzymes, microbial communities and plant roots. AMB Express.

[32] Mahmood A, Turgay OC, Farooq M, Hayat R (2016). Seed biopriming with plant growth promoting rhizobacteria: a review. FEMS Microbiol. Ecol..

[33] Mortlock MY, Vanderlip RL (1989). Germination and establishment of pearl millet and sorghum of different seed qualities under controlled high-temperature environments. Field Crops Res..

[34] Bond JJ, Army TJ, Lehman OR (1964). Row spacing, plant populations and moisture supply as factors in dryland grain sorghum production 1. Agron. J..

[35] Jones OR, Johnson GL (1991). Row width and plant density effects on texas high plains sorghum. J. Prod. Agric..

[36] Faisal M, Barani ARS, Malik A, Hussain M, Awan SI (2007). Yield response of fodder sorghum (Sorghum bicolor) to seed rate and row spacing under rain-fed conditions. J. Agric. Soc. Sci. Pak..

[37] McGuire SJ (2007). Vulnerability in farmer seed systems: Farmer practices for coping with seed insecurity for sorghum in Eastern Ethiopia. Econ. Bot..

[38] Snider JL, Raper RL, Schwab EB (2012). The effect of row spacing and seeding rate on biomass production and plant stand characteristics of non-irrigated photoperiod-sensitive sorghum (*Sorghum bicolor* (L.) Moench). Ind. Crops Prod..

[39] Place GT, Reberg-Horton SC, Dunphy JE, Smith AN (2009). Seeding rate effects on weed control and yield for organic soybean production. Weed Technol..

[40] Harvey TL, Thompson CA (1988). Effects of sorghum density and resistance on infestations of Greenbug, Schizaphis graminum (Homoptera: Aphididae). J. Kans. Entomol. Soc..

[41] Riedell WE (2010). Mineral-nutrient synergism and dilution responses to nitrogen fertilizer in field-grown maize. J. Plant Nutr. Soil Sci..

[42] Pii Y, Cesco S, Mimmo T (2015). Shoot ionome to predict the synergism and antagonism between nutrients as affected by substrate and physiological status. Plant Physiol. Biochem..

[43] Rietra RPJJ, Heinen M, Dimkpa CO, Bindraban PS (2017). Effects of Nutrient antagonism and synergism on yield and fertilizer use efficiency. Commun. Soil Sci. Plant Anal..

[44] Santos EF, Pongrac P, Reis AR, White PJ, Lavres J (2018). Phosphorus–zinc interactions in cotton: consequences for biomass production and nutrient-use efficiency in photosynthesis. Physiol. Plant..

[45] Egamberdiyeva D (2007). The effect of plant growth promoting bacteria on growth and nutrient uptake of maize in two different soils. Appl. Soil Ecol..

[46] Bindraban PS, Dimkpa C, Nagarajan L, Roy A, Rabbinge R (2015). Revisiting fertilisers and fertilisation strategies for improved nutrient uptake by plants. Biol. Fertil. Soils.

[47] Yahya A (1998). Salinity effects on growth and on uptake and distribution of sodium and some essential mineral nutrients in sesame. J. Plant Nutr..

[48] Alam S, Kamei S, Kawai S (2001). Effect of iron deficiency on the chemical composition of the xylem sap of barley. Soil Sci. Plant Nutr..

[49] Wei Yang TJ, Perry PJ, Ciani S, Pandian S, Schmidt W (2008). Manganese deficiency alters the patterning and development of root hairs in Arabidopsis. J. Exp. Bot..

[50] Dimkpa CO (2015). ZnO nanoparticles and root colonization by a beneficial pseudomonad influence essential metal responses in bean (*Phaseolus vulgaris*). Nanotoxicology.

[51] Petti C, Hirano K, Stork J, DeBolt S (2015). Mapping of a cellulose-deficient mutant named dwarf1-1 in sorghum bicolor to the green revolution gene gibberellin20-oxidase reveals a positive regulatory association between gibberellin and cellulose biosynthesis. Plant Physiol..

[52] Xia Y, Greissworth E, Mucci C, Williams MA, Bolt SD (2013). Characterization of culturable bacterial endophytes of switchgrass (*Panicum virgatum* L.) and their capacity to influence plant growth. GCB Bioenergy.

[53] Chaney AL, Marbach EP (1962). Modified reagents for determination of urea and ammonia. Clin. Chem..

[54] Fiske CH, Subbarow Y (1925). The colorimetric determination of phosphorus. J. Biol. Chem..

[55] Miller GL, Dickens R (1996). Bermudagrass carbohydrate levels as influenced by potassium fertilization and cultivar. Crop Sci..

[56] Serson W (2020). Development of whole and ground seed near-infrared spectroscopy calibrations for oil, protein, moisture, and fatty acids in *Salvia hispanica*. J. Am. Oil Chem. Soc..

[57] Saleem M, Law AD, Moe LA (2016). Nicotiana roots recruit rare rhizosphere taxa as major root-inhabiting microbes. Microb. Ecol..

[58] Meng L (2019). Soil-applied biochar increases microbial diversity and wheat plant performance under herbicide fomesafen stress. Ecotoxicol. Environ. Saf..

[59] Mounde LG, Boh MY, Cotter M, Rasche F (2015). Potential of Rhizobacteria for promoting sorghum growth and suppressing Striga hermonthica development. J. Plant Dis. Prot..

[60] Kumar H, Dubey RC, Maheshwari DK (2017). Seed-coating fenugreek with Burkholderia rhizobacteria enhances yield in field trials and can combat Fusarium wilt. Rhizosphere.

[61] Vandenkoornhuyse P, Quaiser A, Duhamel M, Van AL, Dufresne A (2015). The importance of the microbiome of the plant holobiont. New Phytol..

[62] Singh M (2015). Complementarity among plant growth promoting traits in rhizospheric bacterial communities promotes plant growth. Sci. Rep..

[63] Lei SA (2004). Intraspecific competition among blackbrush (*Coleogyne ramosissima*) seedlings in a controlled environmental glasshouse. J. Ariz.-Nev. Acad. Sci..

[64] XiaoAn Z (2013). Seasonal changes in the relationship between species richness and community biomass in grassland under grazing and exclosure, Horqin Sandy Land, northern China. Sci. Cold Arid Reg..

[65] de Aguiar MI, Fialho JS, de Araújo FCS, Campanha MM, de Oliveira TS (2013). Does biomass production depend on plant community diversity?. Agrofor. Syst..

[66] Falzari LM, Menary RC, Dragar VA (2006). Optimum stand density for maximum essential oil yield in commercial fennel crops. HortScience.

[67] Ghiasy-Oskoee M, AghaAlikhani M, Mokhtassi-Bidgoli A, Sefidkon F, Ayyari M (2019). Seed and biomass yield responses of blessed thistle to nitrogen and density. Agron. J..

[68] Isaac ME, Ulzen-Appiah F, Timmer VR, Quashie-Sam SJ (2007). Early growth and nutritional response to resource competition in cocoa-shade intercropped systems. Plant Soil.

[69] Blank RR (2010). Intraspecific and interspecific pair-wise seedling competition between exotic annual grasses and native perennials: plant–soil relationships. Plant Soil.

[70] Dobermann, A. R. *et al.* Understanding and Managing Corn Yield Potential. *Agron. Hortic. -- Fac. Publ.* (2002).

[71] Sabais ACW (2012). Soil organisms shape the competition between grassland plant species. Oecologia.

[72] Munoz AE, Weaver RW (1999). Competition between Subterranean Clover and Rygrass for uptake of 15N-labeled fertilizer. Plant Soil.

[73] Eisenhauer N, Scheu S (2008). Invasibility of experimental grassland communities: the role of earthworms, plant functional group identity and seed size. Oikos.

[74] Tesfaye M (2003). Influence of enhanced malate dehydrogenase expression by alfalfa on diversity of rhizobacteria and soil nutrient availability. Soil Biol. Biochem..

[75] Fernandez AL (2016). Associations between soil bacterial community structure and nutrient cycling functions in long-term organic farm soils following cover crop and organic fertilizer amendment. Sci. Total Environ..

[76] Bashan Y, Holguin G, de-Bashan LE (2004). Azospirillum-plant relationships: physiological, molecular, agricultural, and environmental advances (1997–2003). Can. J. Microbiol..

[77] Dinesh R (2013). Effects of plant growth-promoting rhizobacteria and NPK fertilizers on biochemical and microbial properties of soils under ginger (*Zingiber officinale*) Cultivation. Agric. Res..

[78] Li Q (2018). Belowground interactions impact the soil bacterial community, soil fertility, and crop yield in maize/peanut intercropping systems. Int. J. Mol. Sci..

[79] Maron P-A (2018). High microbial diversity promotes soil ecosystem functioning. Appl. Environ. Microbiol..

[80] Loreau M, Naeem S, Inchausti P, Loreau M, Naeem S, Inchausti P (2002). Biodiversity and ecosystem functioning: synthesis and perspectives. Biodiversity and Ecosystem Functioning: Synthesis and Perspectives.

[81] Patten CL, Glick BR (2002). Role of pseudomonas putida Indoleacetic Acid in development of the host plant root system. Appl. Environ. Microbiol..

[82] Compant S, Clément C, Sessitsch A (2010). Plant growth-promoting bacteria in the rhizo- and endosphere of plants: their role, colonization, mechanisms involved and prospects for utilization. Soil Biol. Biochem..

[83] Sahn DE (2015). The Fight Against Hunger and Malnutrition: The Role of Food, Agriculture, and Targeted Policies.

[84] Schmidt SB, Jensen PE, Husted S (2016). Manganese deficiency in plants: the impact on photosystem II. Trends Plant Sci..

[85] Lambers H, Hayes PE, Laliberté E, Oliveira RS, Turner BL (2015). Leaf manganese accumulation and phosphorus-acquisition efficiency. Trends Plant Sci..

[86] de Santiago A, Quintero JM, Avilés M, Delgado A (2011). Effect of Trichoderma asperellum strain T34 on iron, copper, manganese, and zinc uptake by wheat grown on a calcareous medium. Plant Soil.

[87] Rajkumar M, Sandhya S, Prasad MNV, Freitas H (2012). Perspectives of plant-associated microbes in heavy metal phytoremediation. Biotechnol. Adv..

[88] Gyaneshwar P, Naresh Kumar G, Parekh LJ, Poole PS (2002). Role of soil microorganisms in improving P nutrition of plants. Plant Soil.

[89] Kuo S, Mikkelsen DS (1981). Effect of P and Mn on growth response and uptake of Fe, Mn and P by sorghum. Plant Soil.

[90] Shri PU, Pillay V (2017). Excess of soil zinc interferes with uptake of other micro and macro nutrients in *Sorghum bicolor* (L.) plants. Indian J. Plant Physiol..

[91] Slaton NA, Roberts TL, Golden BR, Ross WJ, Norman RJ (2013). Soybean response to phosphorus and potassium supplied as inorganic fertilizer or poultry litter. Agron. J..

[92] Griffin EA, Wright SJ, Morin PJ, Carson WP (2017). Pervasive interactions between foliar microbes and soil nutrients mediate leaf production and herbivore damage in a tropical forest. New Phytol..

[93] Harpole WS (2011). Nutrient co-limitation of primary producer communities. Ecol. Lett..

[94] Zuo Y, Zhang F (2011). Soil and crop management strategies to prevent iron deficiency in crops. Plant Soil.

