# Supplementary material for: Rhizobacterial species richness improves sorghum growth and soil nutrient synergism in a nutrient-poor greenhouse soil
Source: Sci Rep. 2020 Sep 22;10:15454. doi: 10.1038/s41598-020-72516-3 (PMC7509789; doi:10.1038/s41598-020-72516-3)
Supplement: Supplementary file 1 — Supplementary Information 1. [file 41598_2020_72516_MOESM1_ESM.docx]

**Supplementary information**

**Rhizobacterial species richness improves sorghum growth and soil nutrient synergism in a nutrient-poor greenhouse soil**

***Affiliations:***

*Mohammad Radhi Sahib ^1,2×^, Zahida H Pervaiz^4^, Mark A. Williams^1^, Muhammad Saleem*^3×^, and Seth DeBolt*^1*^

^1^Department of Horticulture, University of Kentucky, Lexington, KY 40546-0312

^2^Department of Horticulture, Al-Qasim Green University, Babylon, Iraq

^3^ Department of Biological Sciences, Alabama State University, Montgomery, Al 36101, USA

^4^ Department of Biological Sciences, Auburn University, Al 36101, USA

*^×^* These authors contributed equally

***Correspondence***:

*Muhammad Saleem, email: msaleem@alasu.edu

Journal: *Scientific Reports*

**Method for determination of plant tissue N and P *as provided by analysis-performing laboratory***

The plant samples were processed for nutrient analysis by weighing 100 mg of dried plant material into 25x200 Pyrex glass ignition tubes that were marked at 50 ml. The five ml of concentrated sulfuric acid that contained 0.05g of salicylic acid per milliliter was added, and then these samples were allowed to react for about one hour at the room temperature (70°F= 21.1°C). This step made any existing inorganic nitrate present in the sample to form the nitrosalicylic acid. Then, 0.5g of sodium thiosulfate was mixed, and these samples were placed in the Technicon BD-40 block digestor at the 180°C for one hour. This resulted in the reduction of nitrosalicylic acid to the less refractory compound named as the aminosalicylic acid. Next, 1.8g of potassium sulfate and 4 selenized boiling chips were added, and then the digestion was continued for 2.5 hours at the 360°C. During this process, all forms of nitrogen were converted to the ammonium, while all forms of the phosphorus were converted to the orthophosphate. Then, these samples were allowed to cool down and then diluted to 50 ml with the deionized water. After mixing thoroughly, these samples were poured into the polystyrene cups for the analysis. The instrument used for the colorimetric measurement of the total nitrogen and total phosphate was the dual Technicon System Il Autoanalyzer that was configured to determine both nitrogen and total phosphate simultaneously. The wavelength was 660 nm for each procedure. The method for the ammonia was a modification of the Bert helot reaction developed by Chaney and Marbach (1962). The two reagents were necessary. One contained 0.5% sodium hydroxide and 0.042% sodium hypochlorite in the de-ionized water; the other contained 1.0 phenol and 0.02% sodium nitroprusside in de-ionized water. The samples were planned into the bubble segmented stream followed by the reagents. The reaction took place inside the instrument, while the blue indophenol formed was passed through the colorimeter for the final measurement of the ammonia concentration. The manual method was modified to accelerate the rate of reaction in order to make it compatible with constraints of the automated system. It was important to enhance the nitroprusside catalyst concentrations to four times, which were recommended in original manual method and to pass the reaction stream through a heating bath set at 60°C. The reagent/sample ratios were kept similar to manual procedure. The acid in the sample was neutralized by adding sodium hydroxide in a dilution loop before the addition of the reagents. The phosphorus technique was according to the method of Fiske and

Subbarow (1925), which is almost same as the Technicon

Industrial Method 348 R 6-3 1-5 except the dialysis step was not necessary for the digested samples. The solution of ammonium molybdate (7 .5g/1000 n l) in 1.92 N sulfuric acid was added and reacted with the sample in the segmented stream to form the heteropoly phosphomolybdate complex. This compound was then reduced by adding the solution containing 150g of sodium bisulfite (NaHS03), 5.0g sodium sulfite and 2.5g 1-Amino-2-Naphthol-4-Sulfonic Acid in the 1000 ml de-ionized water, and then heated the reagent stream up to 95°C in the oil bath. The reaction caused the formation of an intense blue color proportional to the the phosphate concentration. Standards were prepared by using a blank solution made by the first digesting a number of 100 mg pieces of cellulose filter paper according to the above-mentioned protocol. The appropriate amounts of ammonium sulfate and sodium dibasic phosphate were then added to make solutions corresponding to 2-8 % N and 0.2-0.8%P. The Technicon system was comprised of peaks on a strip chart recorder. Four standards and a blank were run before and after each set of 15 samples, and the average was used to make the calculation to minimize errors caused by the baseline drift. The peaks were digitized using a graphics tablet (Summagraphics), and the transferred to a spreadsheet program (SigmaScan). The Macros written " in house" were used to determine the nitrogen and phosphorus concentrations by linear interpolation.

**Method for determination of plant tissue K contents**

We determined potassium (K) contents of plat tissue following acid- digest determination. Briefly, we grounded plant tissue samples (upto 2mm). Then, we weigh and put 250 mg plant samples into 50 mL flask. These flasks were placed in the muffle furnace (thermolyne 30400) overnight at 500 C. After cooling, we add 2 mL of conc. HCL (12.1 N) swirled it. Then, we added 23 mL of water to make this solution with acid concentration of 1N HCL. The samples were heated on steam plate cover with watch glass or marble for 20 minutes. After cooling, we made dilutions and standards using a Hamilton digital diluter. Then, we run these samples on Atomic Absorption Spectrometer SpectrAA 50B for measuring %K contents.

Important note: The above-mentioned method of N,P, and K analysis is provided by the lab that analyzed our samples while authors are not aware of any copyright issues. But nevertheless, we have provided the relevant references as we found in the literature^1–6^.

**References:**

1. Serson, W. *et al.* Development of Whole and Ground Seed Near-Infrared Spectroscopy Calibrations for Oil, Protein, Moisture, and Fatty Acids in Salvia hispanica. *Journal of the American Oil Chemists’ Society* **97**, 3–13 (2020).

2. Chaney, A. L. & Marbach, E. P. Modified Reagents for Determination of Urea and Ammonia. *Clin Chem* **8**, 130–132 (1962).

3. Bradstreet, R. B. Kjeldahl method for organic nitrogen. *Analytical Chemistry* **26**, 185–187 (1954).

4. Sm, D., Wl, B. & Mc, G. Studies on the phospholipid requirement of glucose 6-phosphatase. *J Biol Chem* **243**, 2216–2228 (1968).

5. Fiske, C. H. & Subbarow, Y. The Colorimetric Determination of Phosphorus. *J. Biol. Chem.* **66**, 375–400 (1925).

6. ALASWAD, F. A. M.Distribution, deposition and spatial variability of carbon in soil using carbon and nitrogen stable isotopes. PhD thesis. (2018). <http://psasir.upm.edu.my/id/eprint/76224/1/FPAS%202018%2018%20-%20IR.pdf>

**Properties of greenhouse soil (potting mix) that we used in our experiments, as reported by the provider.**
