# Supplementary material for: Rhizobacterial species richness improves sorghum growth and soil nutrient synergism in a nutrient-poor greenhouse soil
Source: Sci Rep. 2020 Sep 22;10:15454. doi: 10.1038/s41598-020-72516-3 (PMC7509789; doi:10.1038/s41598-020-72516-3)
Supplement: Supplementary file 2 — Supplementary Information 2. [file 41598_2020_72516_MOESM2_ESM.pdf]

Supplementary information

Rhizobacterial species richness improves sorghum growth and soil nutrient synergism in a nutrient-poor greenhouse soil

Affiliations:

Mohammad Radhi Sahib [1,2×], Zahida H Pervaiz[4], Mark A. Williams[1], Muhammad Saleem[3×], and Seth DeBolt*[1*]

[1]Department of Horticulture, University of Kentucky, Lexington, KY 40546-0312

[2]Department of Horticulture, Al-Qasim Green University, Babylon, Iraq

[3] Department of Biological Sciences, Alabama State University, Montgomery, Al 36101, USA

[4] Department of Biological Sciences, Auburn University, Al 36101, USA

[×] These authors contributed equally

Correspondence:

*Muhammad Saleem, email: msaleem@alasu.edu

Journal: *Scientific Reports*

Supplementary figures

Figure S1

Impact of individual rhizobacterial species and their various combinations on seed germination. The statistical differences as indicated by letters were determined by ANOVA followed by Fisher's post hoc test.

Figure S2

Impact of individual rhizobacterial species and their various combinations on the shoot dry mass (a) number of root branches (b), root biomass per pot (c), shoot biomass per plant (d), root branches per plant (e) and root biomass per plant (f). The statistical differences as indicated by letters were determined by ANOVA followed by Fisher's post hoc test.

Figure S3

Relationship of the plant density with per plant shoot biomass (a), root biomass (b), and root branches (c) in the rhizobacterial treatments. The statistical analysis shows linear-regression followed by ANOVA describing the relationships between these parameters. While describing these relationships, we excluded the control treatments and presented them separately (Fig S4).

Figure S4

Relationship of the plant density with per plant (PP) shoot biomass (a), root biomass (b), and root branches (c) in the control treatments.

Figure S5

Impact of various rhizobacterial combinations on shoot (ace) and root (bdf) nutrient contents. The statistical differences as indicated by letters were determined by ANOVA followed by Fisher's post hoc test.

Figure S6

Relationship of the plant density with shoot (abc) and root (jkl) nutrient contents in the rhizobacterial treatments. The relationship of the per pot plant shoot (def) and root (mno) dry mass with nutrient contents in the rhizobacterial treatments. The relationship of per plant shoot (ghi) and root (pqr) dry mass with nutrient contents in rhizobacterial treatments. The statistical analysis shows linear-regression describing the relationships between these parameters. While describing

these relationships, we excluded the control treatments and presented them separately (Fig. S7).

Figure S7

Relationship of the plant density with shoot (abc) and root (jkl) nutrient contents in the control treatments. The relationship of the per pot plant shoot (def) and root (mno) dry mass with nutrient contents in the control treatments. The relationship of per plant shoot (ghi) and root (pqr) dry mass with nutrient contents in the control treatments.

Figure S8

Impact of individual rhizobacterial species and their various combinations on soil nutrient contents. The statistical differences as indicated by letters were determined by ANOVA followed by Fisher's post hoc test.

Figure S9

The relationship of plant density (a) and root branches (b) with soil Mn contents in the control treatment.

Figure S10

Relationships between various soil macro-and micro-nutrients in the control treatment.

Figure S11

Relationships of some soil nutrients with plant tissue nutrients in the control treatment.

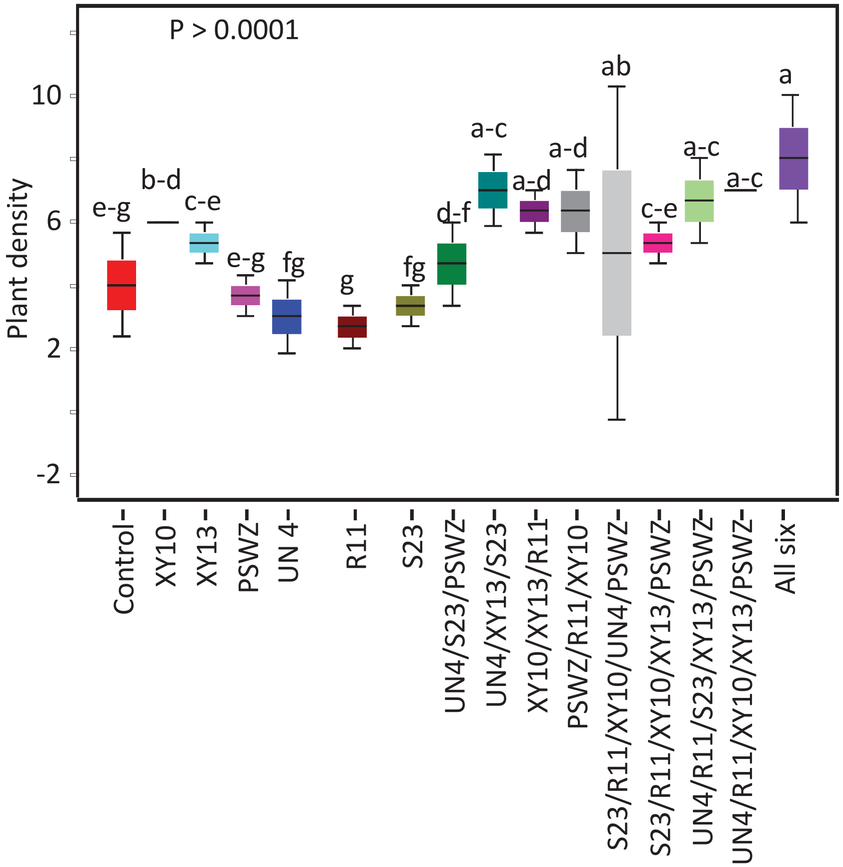

Supporting Figure 1

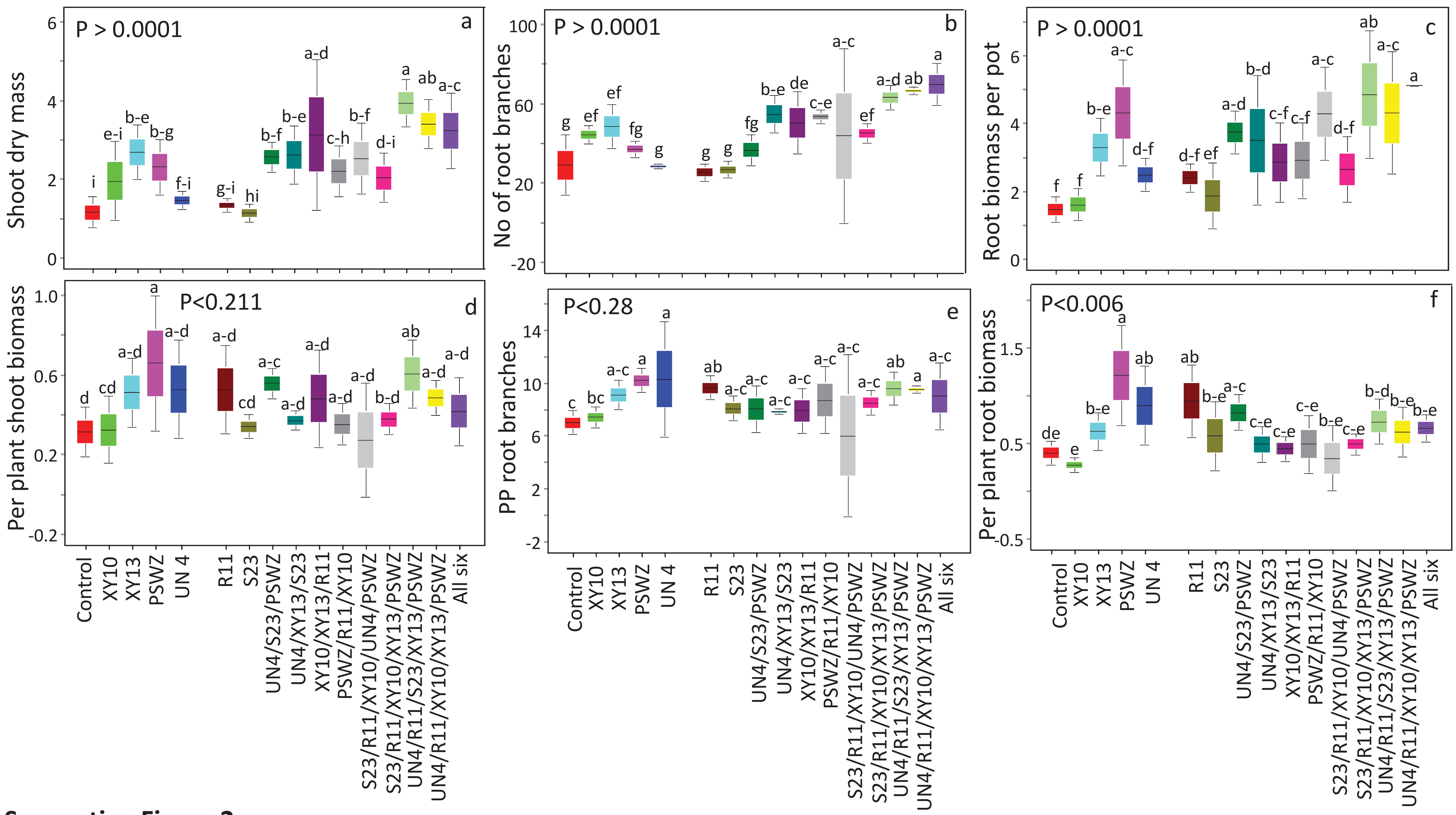

Supporting Figure 2

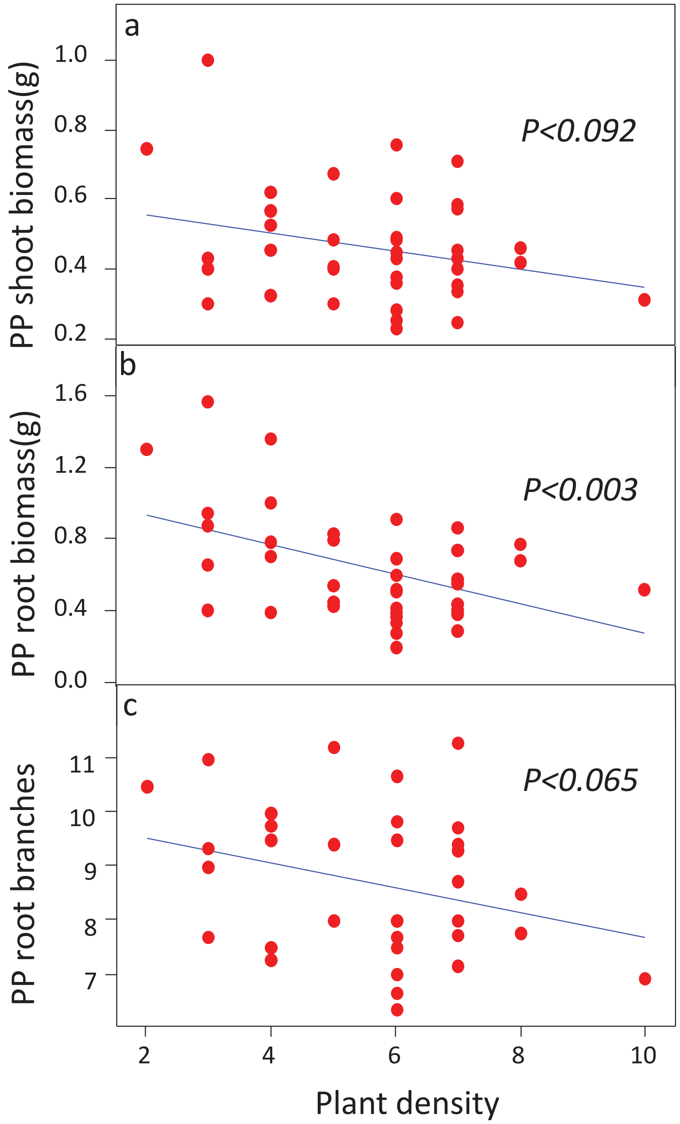

Supporting Figure 3

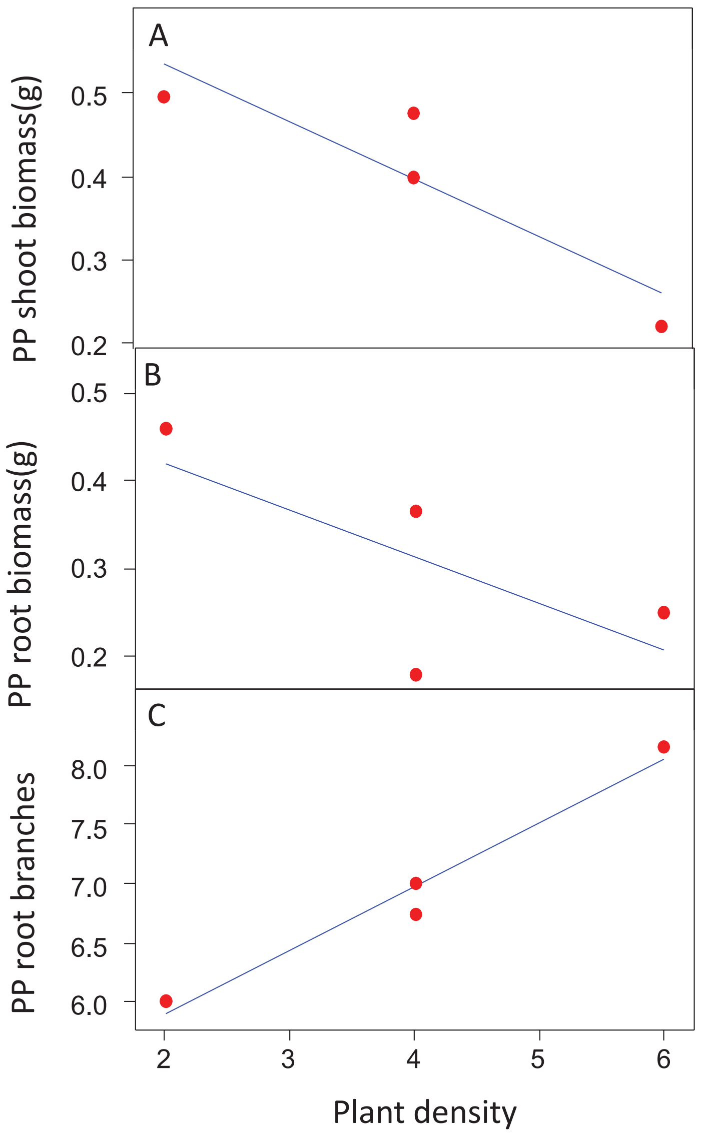

Supporting Figure 4

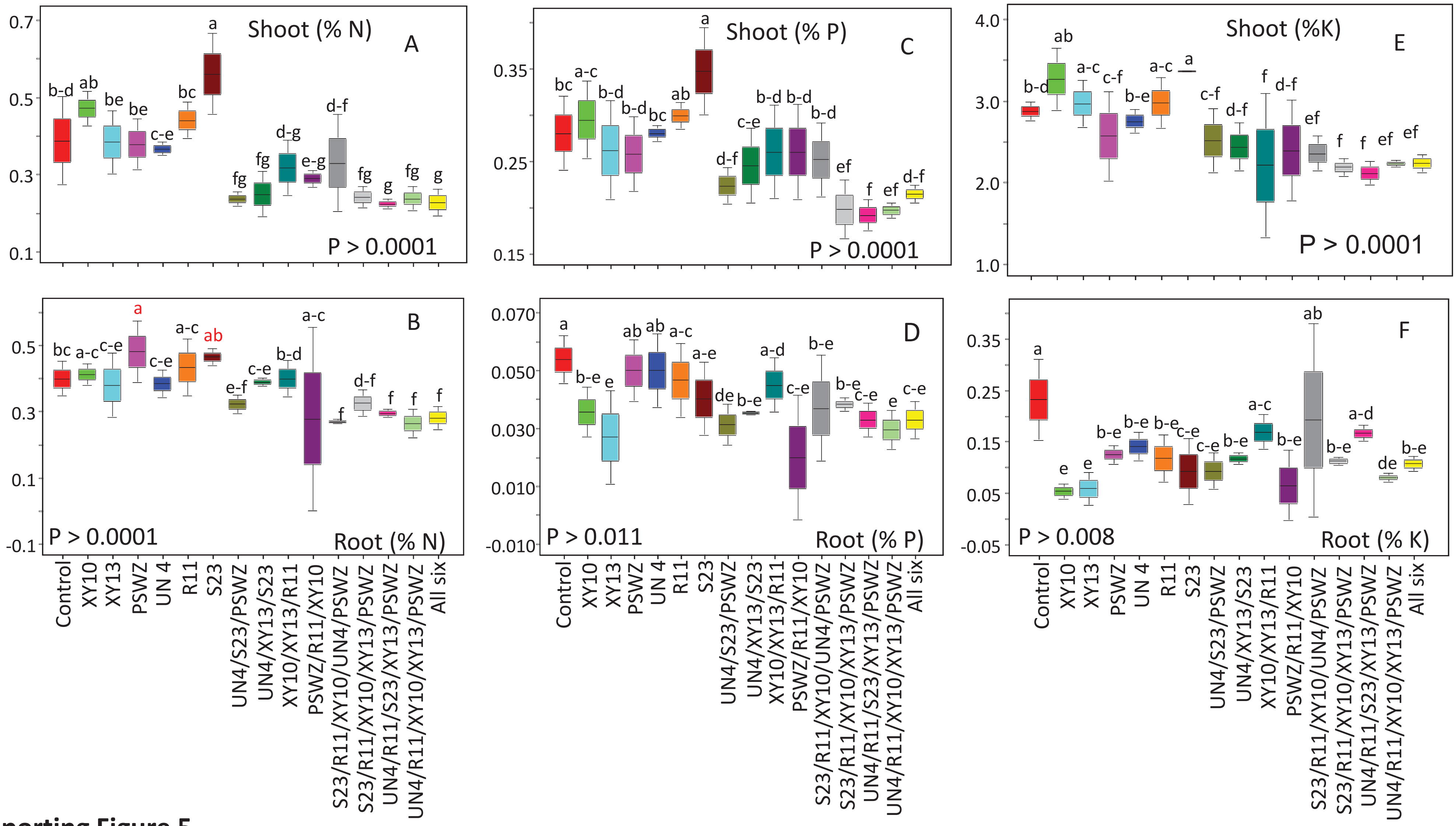

Supporting Figure 5

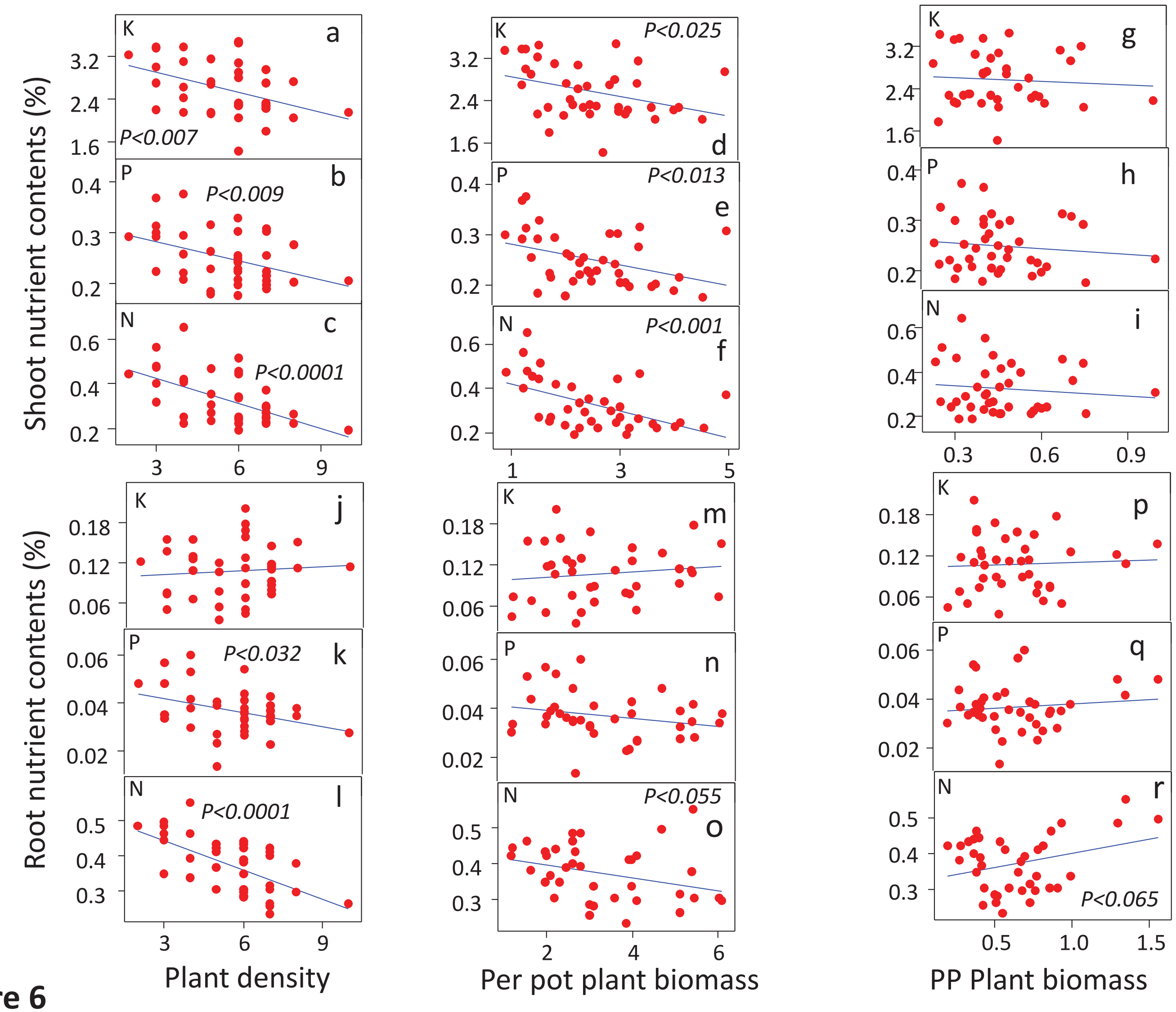

Supporting Figure 6

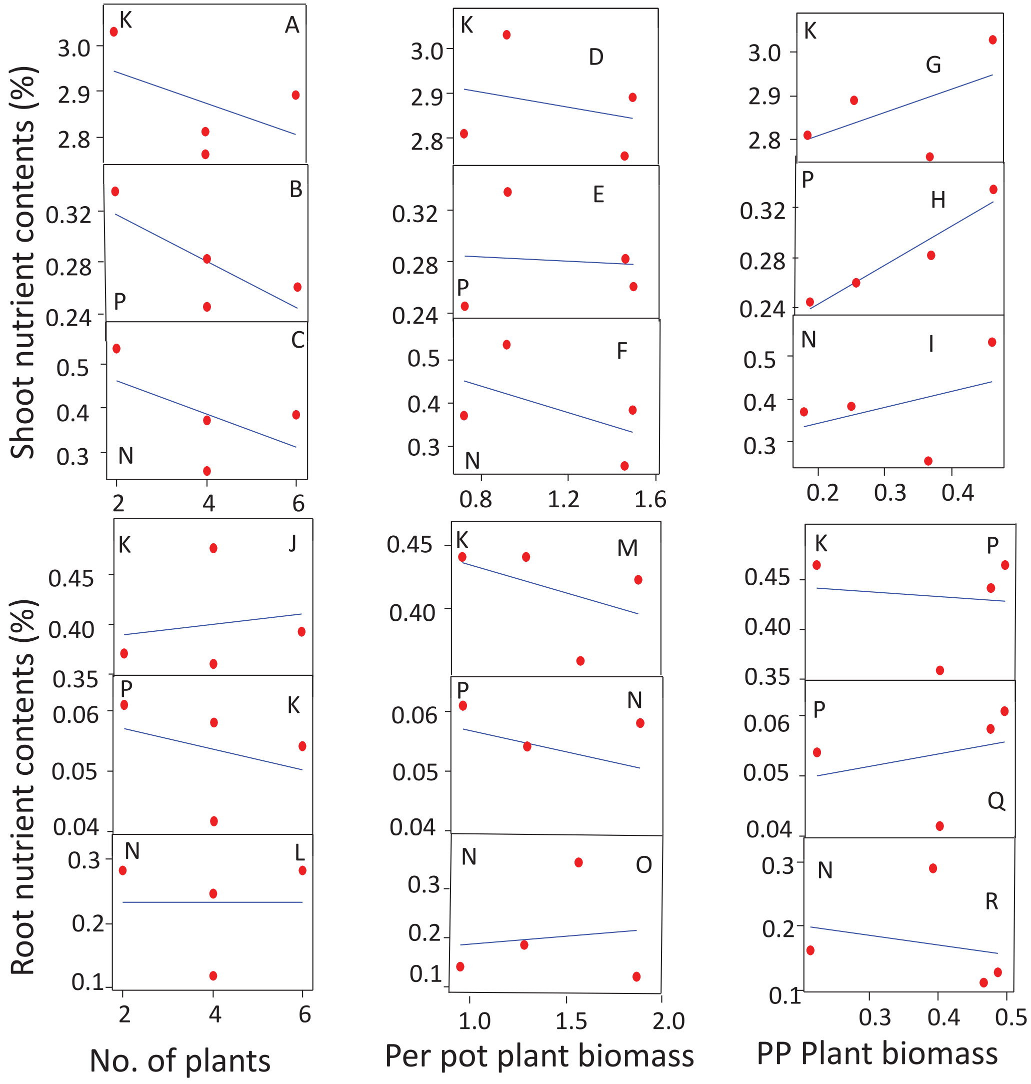
Shoot nutrient contents (%)
Root nutrient contents (%)
A
B
C
D
E
F
G
H
I
J
K
L
M
N
O
P
Q
R
K
P
N
3.0
2.9
2.8
0.32
0.28
0.24
0.5
0.4
0.3
0.45
0.40
0.35
0.06
0.05
0.04
0.3
0.2
0.1
2
4
6
0.8
1.2
1.6
0.2
0.3
0.4
1.0
1.5
2.0
0.5
No. of plants
Per pot plant biomass
PP Plant biomass

Figure S7

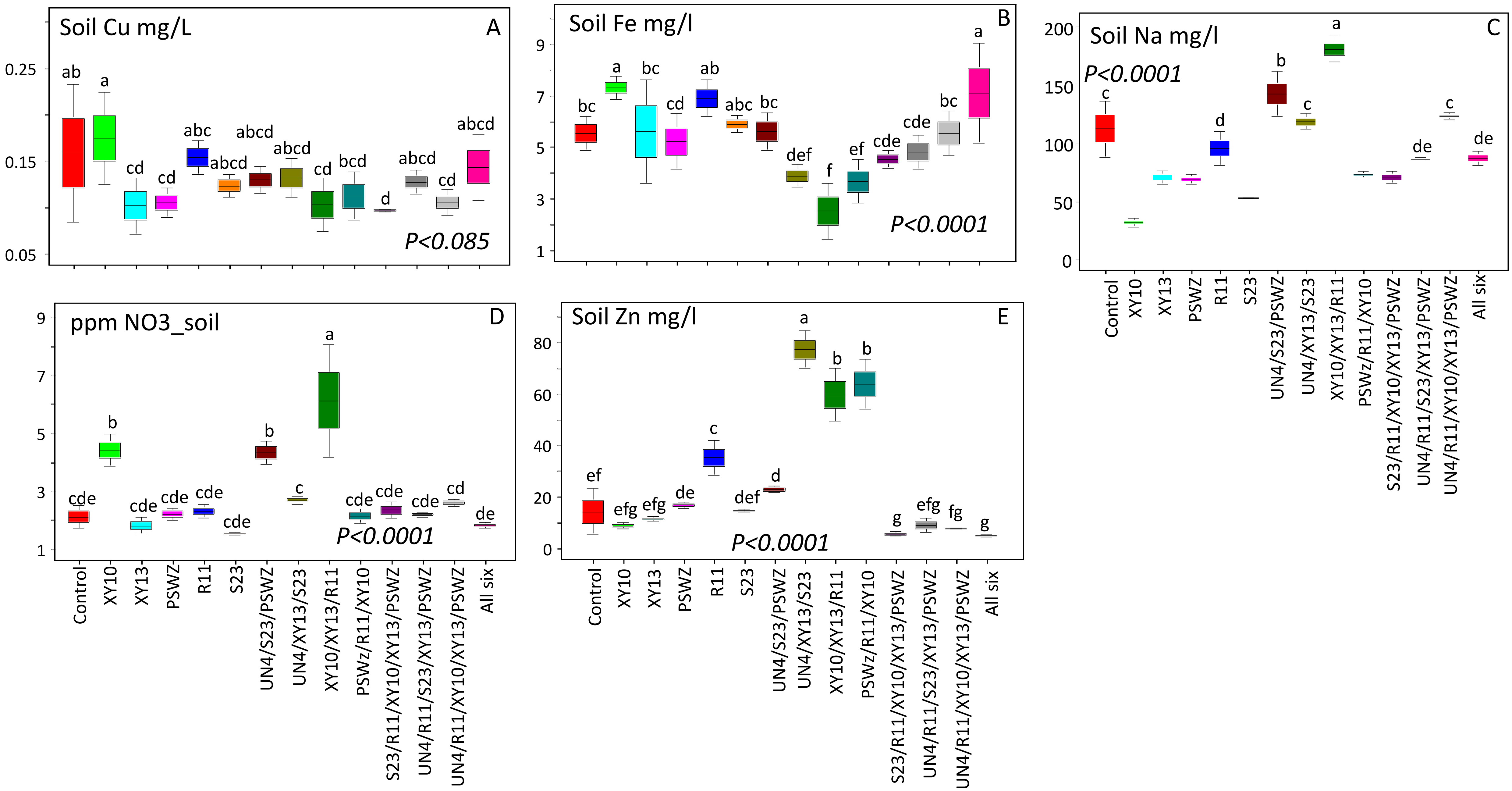

Supporting Figure 8

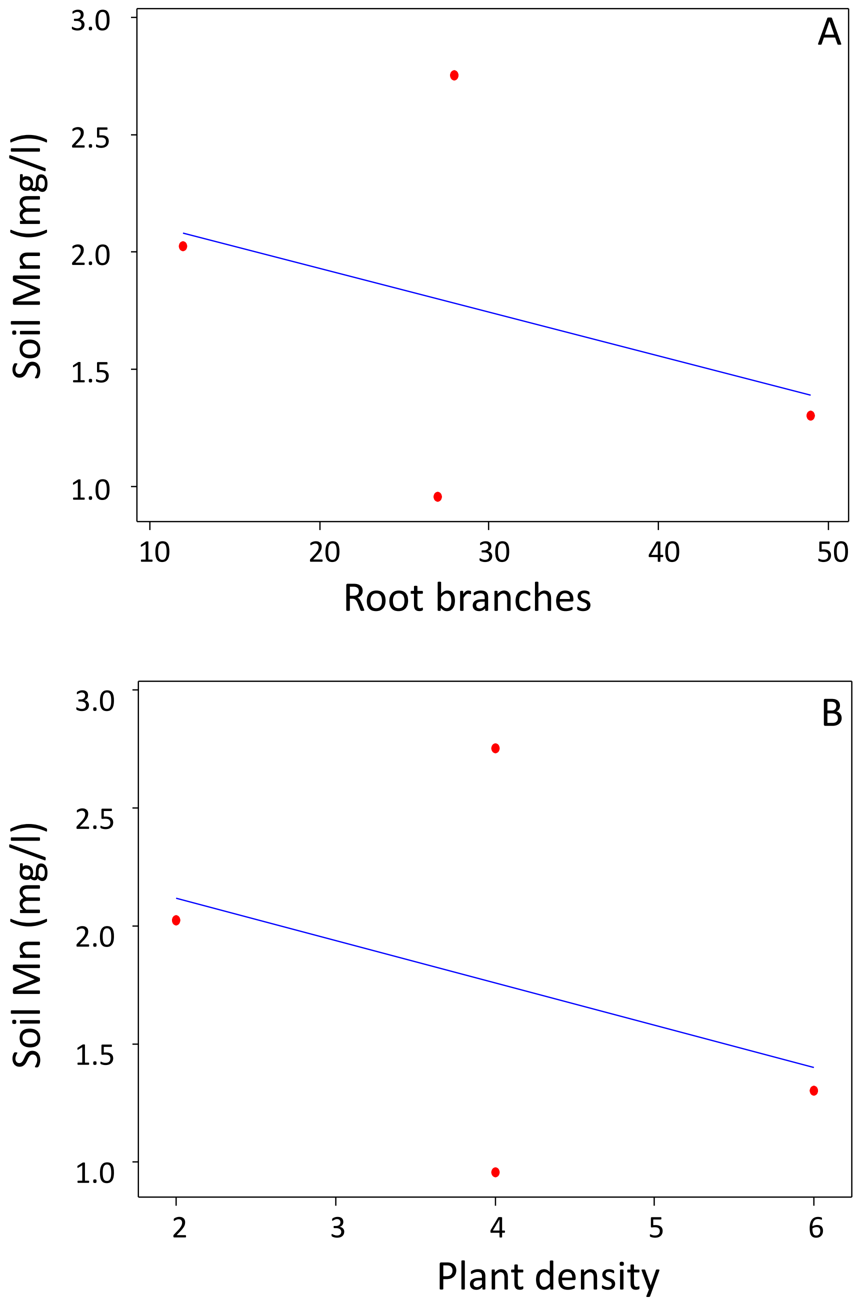

Supporting Figure 9

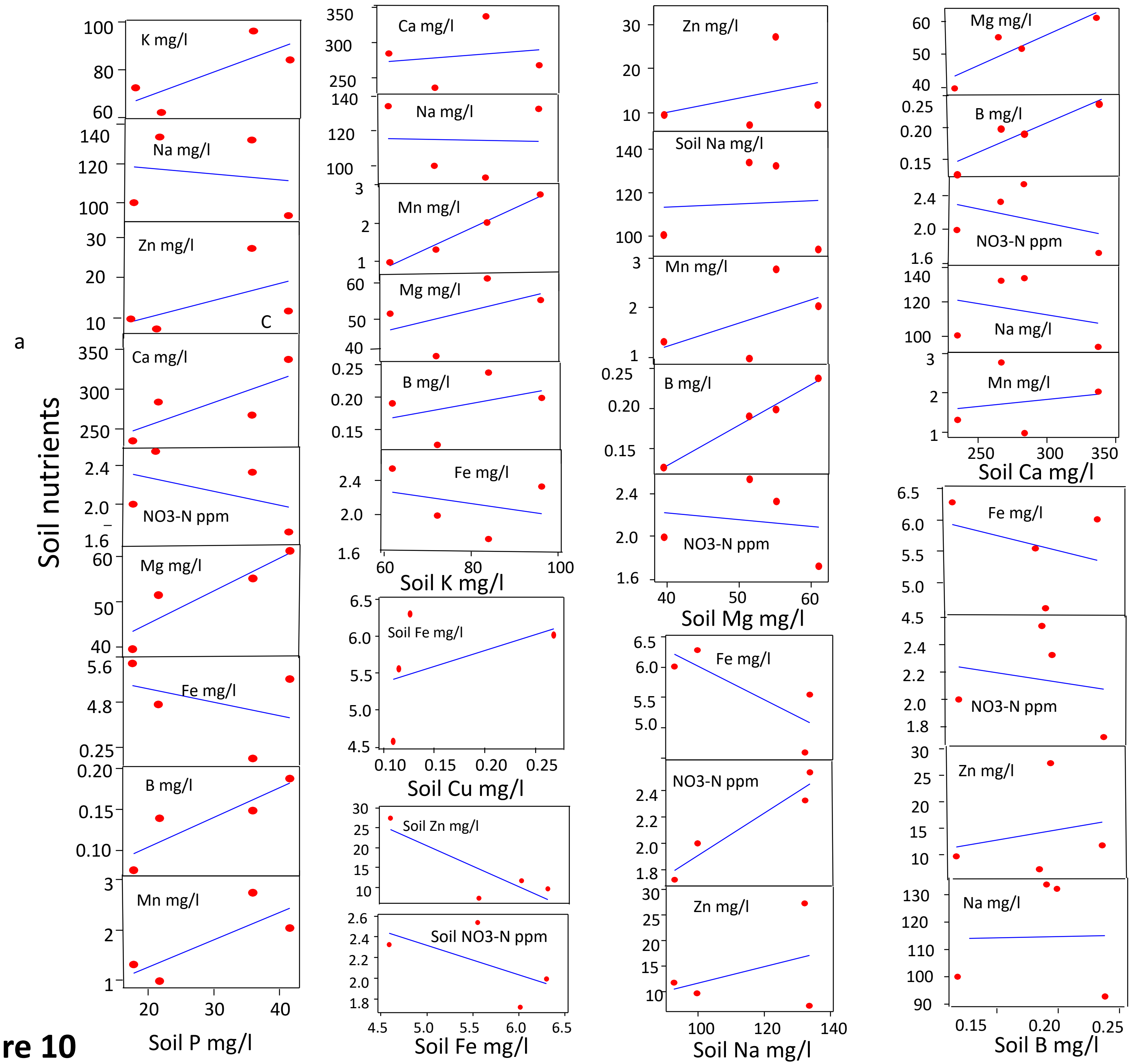

Supporting Figure 10

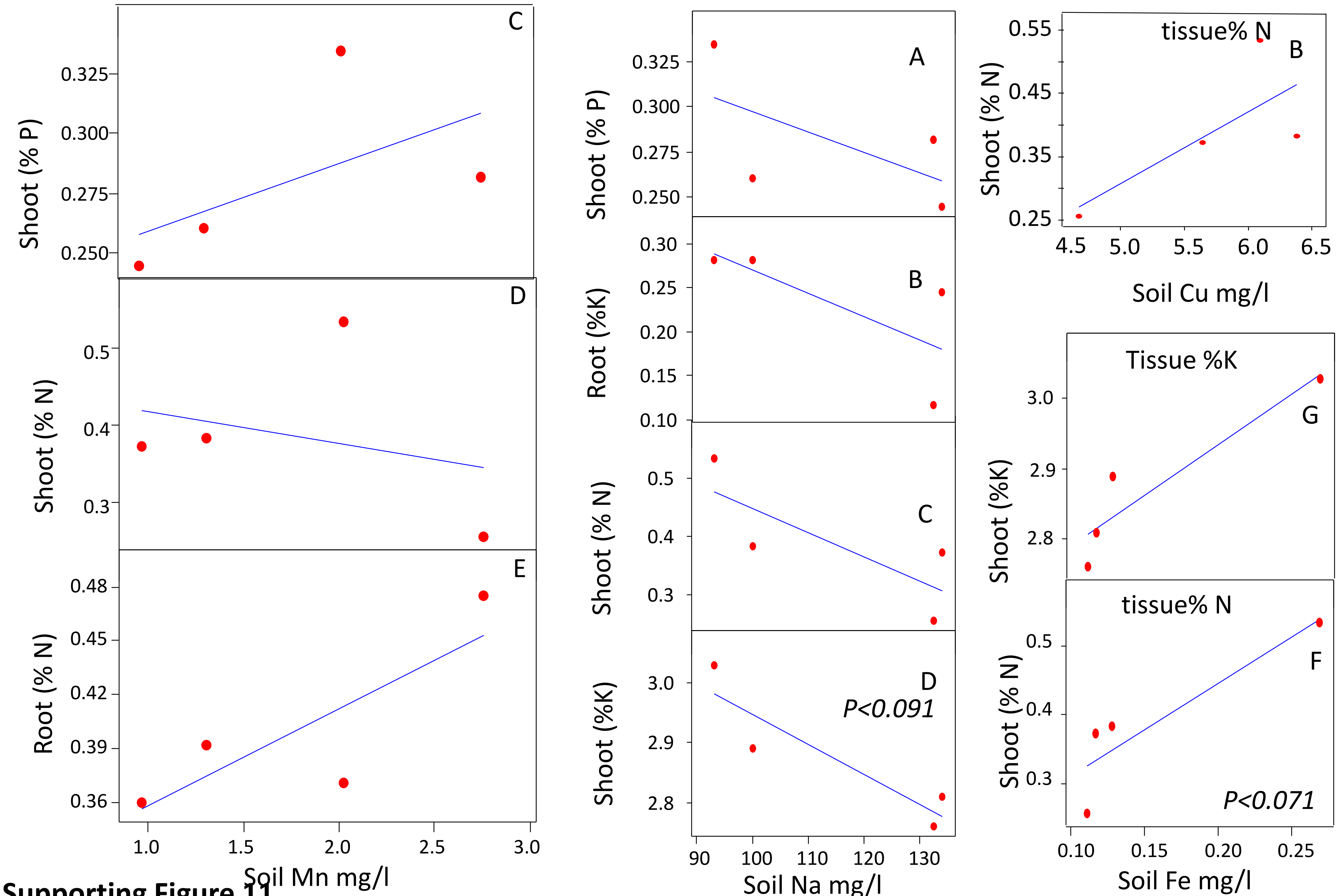

Supporting Figure 11
